# Poly(2-vinylpyridine) as a reference compound for mass calibration in positive-ion matrix-assisted laser desorption/ionization-mass spectrometry on different instrumental platforms

**DOI:** 10.1177/14690667211055701

**Published:** 2021-11-05

**Authors:** Jürgen H Gross

**Affiliations:** Institute of Organic Chemistry, 9144 Heidelberg University, Im Neuenheimer Feld 270, 69120 Heidelberg, Germany

**Keywords:** Matrix-assisted laser desorption/ionization (MALDI), time-of-flight (TOF), ion mobility-quadrupole-time-of-flight (IM-Q-TOF), Fourier transform ion cyclotron resonance (FT-ICR), poly(2-yinylpyridine) (P2VP), accurate mass, mass calibration, small molecules, polymers

## Abstract

Butyl-terminated poly(2-vinylpyridine) (P2VP), C_4_H_9_(C_7_H_7_N)*_n_* H, is evaluated for use as an external and internal mass calibrant in positive-ion matrix-assisted laser desorption/ionization-mass spectrometry (MALDI-MS). P2VP oligomers covering the *m/z* 450–4500 range are employed to calibrate a time-of-flight (TOF) mass spectrometer in linear and reflector mode, an ion mobility-quadrupole-time-of-flight (IM-Q-TOF) mass spectrometer, and a Fourier transform ion cyclotron resonance (FT-ICR) mass spectrometer. The proton affinity of P2VPs introduced by the numerous pyridyl groups leads to the almost exclusive formation of [M + H]^+^ ions with common acidic matrices like α-cyano-4-hydroxycinnamic acid (CHCA) and 2,5-dihydroxybenzoic acid (DHB) as well as with the non-acidic and aprotic matrices 1,8-dihydroxy-10*H*-anthracen-9-on (dithranol) and 2-[(2*E*)-3-(4-*tert*-butylphenyl)-2-methylprop-2-enylidene]malonitrile (DCTB). This prevalence of [M + H]^+^ ions evenly spaced at Δ(*m/z*) = 105.0578 renders butyl-terminated P2VP oligomers as convenient mass calibrants. The mass accuracies achieved across various *m/z* ranges with different mass analyzers and modes of operation are evaluated by using established standard compounds. Results as obtained by internal or external calibration are presented. Further, the compilation of mass reference lists tailored to suit the respective analyzer modes is discussed and those reference files are provided.

## Introduction

Matrix-assisted laser desorption/ionization (MALDI)^1^ is still prevalently implemented in combination with axial time-of-flight (TOF) mass spectrometers, the latest generation of which is capable of providing sufficiently high mass resolving power (*R* = 10 000–25 000) for their use in accurate mass measurement, and thus, molecular formula determination. In contrast to Fourier transform ion cyclotron resonance (FT-ICR) or Orbitrap analyzers, axial TOF analyzers tend to exhibit a drift in mass calibration on the minute-time scale. While these drifts on the minute-time scale are comparatively small on the latest generation instruments, they are still in the order of 5–20 ppm, and thus, preclude or at least restrict the use of external mass calibration for accurate mass measurements. In case of MALDI-MS with axial TOF analyzers, drift in *m/z* is not solely affected by instrumental fluctuations but even more so by local changes in sample layer thickness and matrix crystallization. The goal of accurate mass measurement in MALDI-TOF-MS is thus best achieved by employing an internal mass calibrant.

The level of mass accuracy required to unambiguously derive a molecular formula from a measured accurate mass much depends on the actual *m/z* of the ion, the variety of elements to be considered, and the number of atoms of these elements to be taken into account.^2–5^ For example, depending on the particular restrictions, an unequivocal formula assignment by accurate mass alone may be achieved up to about *m/z* 500^6^ but can become extremely demanding in other situations.^7^ Despite the fact that there is no single generally valid level of mass accuracy to apply to any analytical quest, chemistry journals tend to define and require these limits according to their editors’ own opinion. The Journal of Organic Chemistry published by the American Chemical Society, for example, states “… a found value within 0.003 *m/z* unit of the calculated value of a parent-derived ion … is usually adequate”.^8^ Angewandte Chemie (and other Wiley journals) requires that “high resolution … data should be provided to an accuracy within … ±0.003 of the calculated values.” (whatever the unit maybe).^9^ The Royal Society of Chemistry journals are a bit more conservative in asking that “Exact masses quoted for identification purposes should be accurate to within 5 ppm (EI and CI) or 10 ppm (FAB or LSIMS).”.^10^ To sum it up in brief and simplicity: a mass accuracy of 1 ppm would be desirable, 3 ppm do cover most journals’ (and thus our clients’) requested levels, and 5 ppm still solve many problems, and at times, can be sufficient for publication.

A reference compound intended to be used for mass calibration should have the following properties: (i) it has to yield intensive signals evenly spaced across an ideally wide *m/z* range, (ii) it should essentially provide a single series of peaks to minimize the risk of erroneous peak assignments, (iii) when used as an internal mass calibrant it should not suppress the analyte or, vice versa, be suppressed by the analyte, and (iv) in case of MALDI, it needs to be compatible with the preferred matrix (and ideally with others, too).^11^

To establish mass calibration over a wide *m/z* range in combination with desorption/ionization techniques numerous procedures based on cluster ion series have been described. They all bear the advantage that they are typically generated from readily available compounds additionally offering a year-long shelf life even when stored in ready-to-use solutions. For example, [arginine*_n_* + H]^+^ and [arginine*_n _*− H]^–^ cluster ions can be used for mass calibration in both positive-ion and negative-ion electrospray ionization (ESI), respectively.^12,13^ For use in direct analysis in real time (DART), the ionic liquid (IL) 1-butyl-3-methylimidazolium tricyanomethide delivers ions covering *m/z* 100–4000 in positive-ion and *m/z* 100–2000 in negative-ion DART-MS. The IL provides a wide distribution of cluster ions at Δ(*m/z*) 229.1330 reflecting the mass of the pair of cation and anion.^14,15^ Alternatively, saccharose cluster ions may be employed for mass calibration in positive-ion DART-MS across the *m/z* 100–2000 range.^16^ In MALDI-MS, [Cs*_n_* I*_n_*_–1_]^+^ and [Cs*_n_*_–1_I*_n_*]^–^ cluster ions are also well established for mass calibration. These caesium salt cluster ions of either polarity are effectively formed from caesium triiodide, CsI_3_, in 2-[(2*E*)-3-(4-*tert*-butylphenyl)- 2-methylprop-2-enylidene]malonitrile (DCTB) matrix.^17^ Due to the versatility of the [Cs*_n_* I*_n_*_–1_]^+^ and [Cs*_n_*_–1_I*_n_*]^–^ cluster ion series some variations of this approach have been developed.^18,19^ While generally allowing for their use in positive-ion and negative-ion modes alike, the generation of cluster ions demands their constituents to be present at comparatively high concentration. Mutual interference with analyte ion formation then either tends to cause suppression of analyte ion or calibrant cluster ion generation, thereby often precluding the use of cluster ions as internal mass calibrants. Thus, cluster ion-based mass calibration procedures are generally limited to use for external mass calibration. Additionally, cluster ion series often have the disadvantage of showing a notable decrease of higher-mass cluster ion abundances.

Polymers or branched molecules of defined molecular mass are superior in this regard. In positive- and negative-ion ESI-MS, a mixture composed of ammonium trifluoroacetate, betaine, 2,4,6-tris(heptafluoropropyl)- 1,3,5-triazine, and various symmetrical hexakis-(fluoroalkoxy)-phosphazenes, also known as Agilent Tune Mix, is well established.^20–22^ Agilent Tune Mix may also serve for mass calibration in negative-ion DART-MS.^23^ Other mass calibration procedures for MALDI use poly(ethylene glycols) (PEGs),^24,25^ basic poly(propylene glycols) (Jeffamines)^11^ or monodisperse dendrimers (SpheriCal^™^)^26,27^ for wide range mass calibration.^28^ Dendrimers with a tailored mass defect to avoid interferences with typical organic analyte ions are also available.^29^ Polyalanine has been presented as a calibrant in MALDI-MS as it covers the range *m/z* 1000–4000 in both positive-ion and negative-ion mode.^30^ Unfortunately, it is neither compatible with DCTB matrix^31^ nor does it allow to yield spectra at low laser fluence crucial for optimum resolution in MALDI-TOF-MS.

The first publication dealing with MALDI-MS of poly(2-vinylpyridine) (P2VP) presented the spectrum of a sample of an average molecular weight of about 28 kDa prepared in dithranol matrix doped with sodium chloride.^32^ Other work dealt with P2VP block copolymers^33^ or poly(4-vinylpyridine) (P4VP) with highly polar end groups.^34^ Here, butyl-terminated P2VP, C_4_H_9_(C_7_H_7_N)*_n_* H, (*n* = 4–48), is introduced as a convenient mass calibrant for positive-ion MALDI-MS to be used as an external or internal reference, in combination with common MALDI matrices, and with different mass analyzer configurations.

## Experimental

### Poly(2-vinylpyridine) standards and sample preparation

Three samples of poly(2-vinylpyridine), i.e., PVP-670, PVP-1k, and PVP-2.1k (PSS Polymer Standards Service GmbH, Mainz, Germany), were used without further treatment. These P2VPs are butyl-terminated, and thus, obey the general formula C_4_H_9_(C_7_H_7_N)*_n_* H.

For MALDI sample preparation, stock solutions of the P2VPs at 5 mg ml^–1^ in tetrahydrofuran were prepared and stored at 5 °C until use. These solutions could be used for weeks without change. P2VP stock solutions were admixed to the matrix 2-[(2*E*)-3-(4-*tert*-butylphenyl)-2-methylprop-2-enylidene] malononitrile (DCTB) that was employed as solution at 10 mg ml^–1^ in tetrahydrofuran. The ratio of analyte-to-matrix solution was 1 : 50 and 1 µl of these solutions was used per sample spot on the stainless steel Scout 384 sample plate.

**Figure 1.**
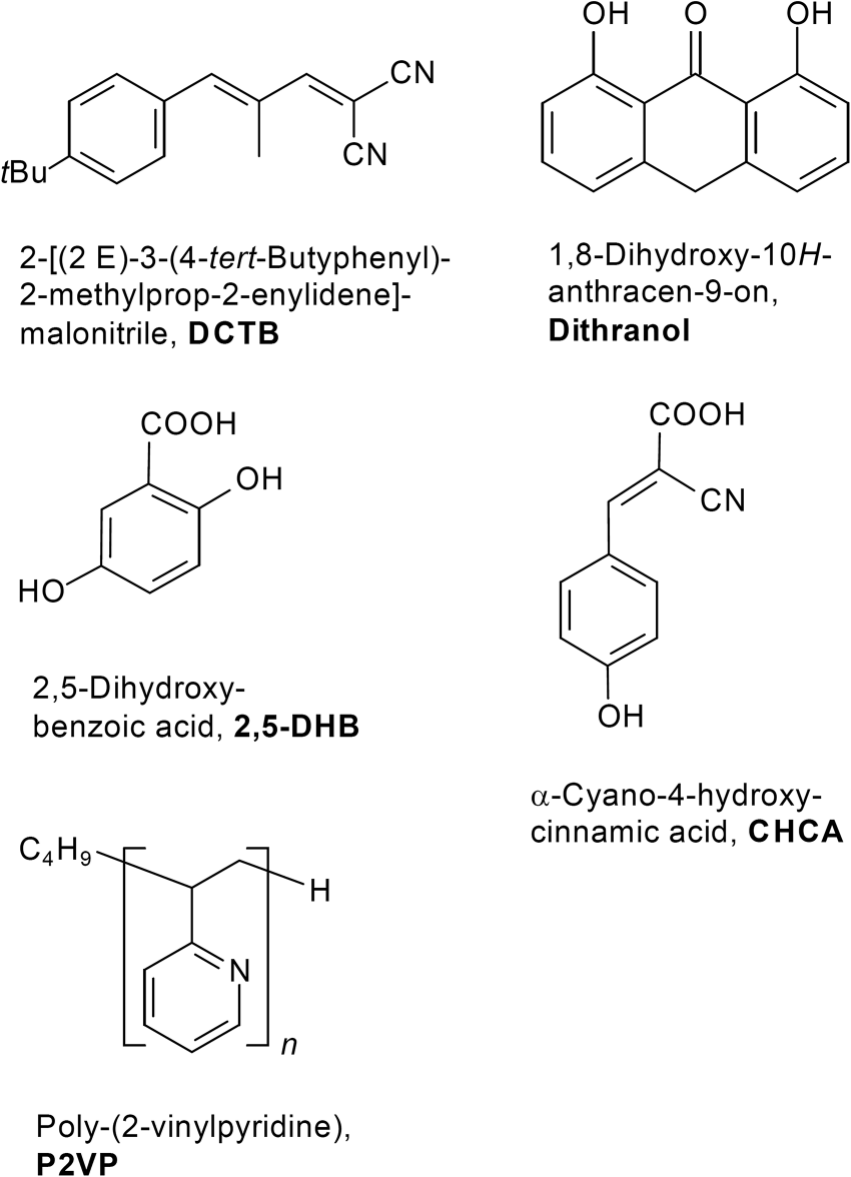
Structures of the matrices 2-[(2*E*)-3-(4-*tert*-butylphenyl)-2-methylprop-2-enylidene] malononitrile (DCTB), 1,8-Dihydroxy-10*H*-anthracen-9-on (dithranol), 2,5-dihydroxybenzoic acid (DHB), α-cyano-4-hydroxycinnamic acid (CHCA), and of the mass calibrant poly(2-vinylpyridine) (P2VP).

For testing the compatibility of P2VPs with the matrices α-cyano-4-hydroxy­cinnamic acid (CHCA), 2,5-dihydroxybenzoic acid (DHB), and 1,8-dihydroxy-10*H*-anthracen-9-on (dithranol), solutions of these matrices were prepared at 10 mg ml^–1^ in acetone. The ratio of analyte-to-matrix solutions was 1 : 50. Structures of the matrices and of P2VP are displayed in Figure 1.

### MALDI-TOF-MS

The MALDI-TOF spectra were acquired using a Bruker Autoflex Speed MALDI-TOF mass spectrometer (Bruker Daltonik GmbH, Bremen, Germany) in both positive-ion linear and reflector mode. The instrument was equipped with a Bruker SmartBeam laser (Nd:YAG, frequency tripled, wavelength 355 nm). It was controlled by Bruker FlexControl 3.4 software and mass spectra were processed using Bruker FlexAnalysis 3.4.

The essential instrument settings for the *m/z* 600–6000 range in linear mode and two typical ranges in reflector mode, i.e., *m/z* 200–2000 and *m/z* 440–5000, are summarized in Table S1. In reflector mode, intermediate ranges like *m/z* 350–3500 were also used as required.

Prior to initial experiments, an independent external mass calibration for positive-ion mode was established based on [Cs*_n_* I*_n_*_–1_]^+^ cluster ions generated from caesium triiodide, CsI_3_, in DCTB matrix.^17^ Mass calibration of the instrument was performed by manual peak assignment to the reference list and application of the cubic enhanced algorithm of Bruker FlexControl 3.4

For use as an internal mass calibrant, the solution of the P2VP best suited for the intended *m/z* range was admixed to the analyte-matrix solution as to achieve relative peak intensities of standard to analyte in the range of 1 : 10 to 3 : 1. The laser fluence was adjusted in order to deliver good intensity signals without sacrificing mass resolution. After assignment of as many as possible reference peaks, internal mass calibration was performed using the cubic enhanced algorithm of Bruker FlexAnalysis 3.4.

### IM-Q-TOF-MS

A Bruker timsTOF flex ion mobility-quadrupole-time-of-flight (IM-Q-TOF) mass spectrometer (Bruker Daltonik GmbH, Bremen, Germany) was used. The instrument was equipped with a Bruker SmartBeam laser (Nd:YAG, frequency tripled, wavelength 355 nm). In MALDI mode the instrument was controlled by the Bruker timsControl software (V 2.0) and data analysis was performed using the Bruker DataAnalysis software (V 5.3). The instrument was set to acquire the *m/z* 250–5000 range. Most relevant settings are summarized in Table S2.

Prior to initial experiments, an independent external mass calibration for positive-ion mode was established based on [Cs*_n_* I*_n_*_–1_]^+^ cluster ions generated from caesium triiodide, CsI_3_, in DCTB matrix.^17^ Mass calibration of the instrument was performed by automatic peak assignment to the reference list and application of the cubic enhanced algorithm of Bruker timsControl software (V 2.0). As this study focuses on *m/z* calibration, the TIMS stage was switched off and IMS was not considered in these experiments.

For use as an external mass calibrant, the solutions of the P2VPs best suited to evenly cover the intended *m/z* range were admixed to the matrix solution, typically PVP-670 and PVP-2.1k at a ratio of 1 : 3. The laser fluence was adjusted in order to deliver good intensity signals without sacrificing mass resolution. After assignment of as many as possible reference peaks, mass calibration was performed using the cubic enhanced algorithm of Bruker timsControl software (V 2.0).

### FT-ICR-MS

A Bruker Apex-Qe Fourier transform ion cyclotron resonance (FT-ICR) mass spectrometer (Bruker Daltonik GmbH, Bremen, Germany) equipped with a 9.4 T superconducting magnet and an ESI-to-MALDI switchable Dual Source MTP was used. The instrument was equipped with a frequency tripled Nd:YAG laser (wavelength 355 nm). The instrument was controlled by the Bruker ApexControl software (V 3.0.0) and data analysis was performed using the Bruker DataAnalysis software (V 4.3).

For MALDI-MS, ions generated by sets of 15–30 laser shots were collected for 0.05 s prior to ICR mass analysis in the RF-only accumulation hexapole (h2). Ions were excited and detected using standard settings from previous work.^23,35,36^ As with the TOF instrument before, an independent external mass calibration for positive-ion mode was established based on [Cs*_n_* I*_n_*_–1_]^+^ cluster ions generated from caesium triiodide, CsI_3_, in DCTB matrix.^17^

In positive-ion DART mode, the IL 1-butyl-3-methylimidazolium tricyanomethide was used for mass calibration prior to analyzing P2VPs.^14,15^ In positive-ion ESI mode, the instrument was externally calibrated using Agilent Tune Mix.^20–22^

Generally, 16–24 transients were accumulated to yield a final FT-ICR mass spectrum. When the range *m/z* 350–3500 was selected, a 1 M data points transient resulted in a resolving power of *R* = 50 000 at *m/z* 1000. When the range *m/z* 740–4500 was selected, a 1 M data points transient resulted in a resolving power of *R* = 95 000 at *m/z* 1000.

## Results and discussion

### General characteristics of P2VP in positive-ion MALDI-MS

**Figure 2.**
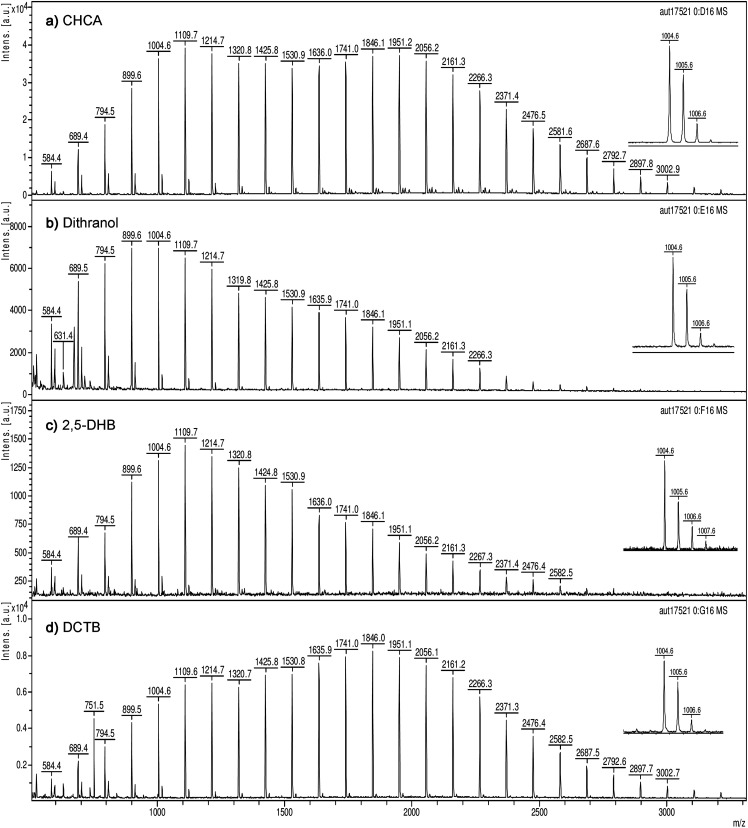
Positive-ion reflector mode MALDI-TOF spectra of PVP-1k in (a) CHCA, (b) dithranol), (c) 2,5-DHB, and (d) DCTB as acquired using the Bruker Autoflex instrument. For comparison of the relative response all spectra were acquired with the laser power set to 45%. The highest intensities in the center of the spectra approximate (a) 4.0 × 10^4^ a.u. (a.u.: arbitrary units of signal intensity), (b) 7.0 × 10^3^ a.u., (c) 1.4 × 10^3^ a.u., and (d) 8.0 × 10^3^ a.u., respectively. The inserts on the right show an expanded view of the signal at *m/z* 1004.6 corresponding to P2VP 9mer [M + H]^+^ ions. Note that the peak picking algorithm labeled the most intensive signal of each of the isotopic patterns, and thus, starting with the 12mer, labels begin referring to those peaks. Further, in case of the 12mer and the 13mer minor fluctuations of relative intensity cause the labels to either refer to the monoisotopic or the first isotopic peak.

First, the three P2VP standards were each admixed to any of the four mentioned matrices (DCTB, CHCA, DHB, dithranol) and the positive-ion MALDI spectra were acquired in both linear and reflector mode of the TOF instrument. In any of these combinations, all P2VPs yield useful spectra that are characterized by the prevalence of just one series of ions (Fig. S1–S8). Based on the reflector mode spectra exhibiting good isotopic separation and with external calibration delivering a mass accuracy in the order of 0.1 u these signals can be assigned to [M + H]^+^ ions. This tentative assignment is, for example, supported by the P2VP 9mer [M + H]^+^ ion, [C_67_H_74_N_9_]^+^ at *m/z* 1004.6 (calc. *m/z* 1004.6061), shown in expanded views of this signal in the spectra of PVP-1k as obtained with any of these matrices (Figure 2). The formation of [M + H]^+^ ions can be expected due to the proton affinity of P2VPs introduced by the numerous pyridyl groups. Advantageously, the almost exclusive formation of [M + H]^+^ ions does not only occur with the acidic matrices CHCA and DHB but also when the non-acidic matrix dithranol and even the aprotic matrix DCTB are employed.

While any of the three P2VPs, together covering the *m/z* 450–5000 range, showed good compatibility with all of these common matrices, there was a notable difference in signal intensity at the same laser fluence. This is demonstrated by comparing the spectra of PVP-1k in (a) CHCA, (b) dithranol), (c) 2,5-DHB, and (d) DCTB as representative of this behavior (Figure 2). With the laser power set to 45% (term used by the software to describe laser fluence) the highest intensities in the center of the spectra approximate (a) 4.0 × 10^4^ a.u. (a.u.: arbitrary units of signal intensity), (b) 7.0 × 10^3^ a.u., (c) 1.4 × 10^3^ a.u., and (d) 8.0 × 10^3^ a.u., respectively. The linear mode spectra exhibit the same tendency. Here PVP-1k in (a) CHCA, (b) dithranol), (c) 2,5-DHB, and (d) DCTB with the laser power set to 25% the highest intensities in the center of the range approximate (a) 2.5 × 10^5^ a.u., (b) 5.0 × 10^3^ a.u., (c) 2.3 × 10^2^ a.u., and (d) 8.0 × 10^4^ a.u., respectively (Fig. S9). Obviously, 2,5-DHB requires the highest laser fluence for effective analyte ion formation followed by dithranol. CHCA and DCTB perform best in that they yield good signal intensities at low laser fluence. To achieve the coverage of any *m/z* range of interest, different P2VPs can simply be mixed (Fig. S10). As DCTB is the most used matrix in this laboratory, unless otherwise noted, the following work is based on DCTB.

To provide an independent confirmation of the formulas of the reference ions to be used in MALDI-MS, the positive-ion DART-FT-ICR spectrum of PVP-670 was acquired using an established procedure (Fig. S11).^14^ While the *m/z* range covered by the DART spectrum with the temperature of the helium gas set to 450 °C did not fully coincide with the *m/z* range observed in MALDI-MS, the spectral data nonetheless confirmed the formulas of a subset of these ions in the central section of the *m/z* range of interest, i.e., from the 4mer [C_32_H_39_N_4_]^+^ at *m/z* 479.3175 (calc. 479.3169) to the 10mer [C_74_H_81_N_10_]^+^ at *m/z* 1109.6665 (calc. 1109.6640). The formula assignments and mass accuracies of all seven ions are also listed in Fig. S11. As the application of DART turned out to be limited to PVP-670, the consistency of the series was additionally checked by measuring the positive-ion ESI-FT-ICR spectrum of PVP-1k in acetonitrile : water : THF = 4 : 2 : 1 with 0.1% trifluoroacetic (Fig. S12). Here, mass calibration was established using Agilent Tune Mix.^20–22^ The ESI-FT-ICR spectrum of PVP-1k covered the 8mer [C_60_H_67_N_8_]^+^ at *m/z* 899.5475 (calc. 899.5483) to the 18mer [C_130_H_137_N_18_]^+^ at *m/z* 1950.1269 (calc. 1950.1268). The formula assignments and mass accuracies of all eleven ions are compiled in Fig. S12. Thus, the assignment of the ion series to P2VP [M + H]^+^ ions was independently confirmed by either DART-FT-ICR-MS or ESI-FT-ICR-MS.

Both the prevalence of [M + H]^+^ ion formation and the occurrence of an ion series evenly spaced at Δ(*m/z*) = 105.0578 as calculated for a C_7_H_7_N monomer unit indicate that P2VPs are potentially convenient mass calibrants.

### Building mass reference lists

Based on these results, a mass reference list can be compiled. As the use of P2VPs is intended in combination with different mass analyzers, one has to take into account that resolving power at a given *m/z* dictates whether isotopic resolution allows for the unambiguous assignment of monoisotopic [M + H]^+^ ions or whether average mass values are required to deal with isotopically unresolved envelopes.

Among the mass analyzers employed in this study, reflector TOF, IM-Q-TOF, and FT-ICR analyzer delivered isotopic resolution beyond *m/z* 5000, and thus, for use as reference values in combination with these, monoisotopic [M + H]^+^ ion masses were calculated (Table 1). Upon growing chain length of the P2VPs, the relative abundances of the monoisotopic ions drop relative to that of the ^13^C_1_, ^13^C_2_, and ^13^C_3_ isotopolog ions, respectively. Therefore, Table 1 provides ^13^C_1_, ^13^C_2_, and ^13^C_3_ isotopolog ion masses and shows some overlap of the four reference *m/z* columns in order to permit proper selection of reference *m/z* values with instruments of resolution characteristics differing from those used in this study. The reference list was found to work best when it provided the *m/z* values calculated for the most abundant isotopolog ion of the respective oligomer (discussion below). Thus, for ions larger than the 11mer, [C_81_H_88_N_11_]^+^, *m/z* 1214.72187 (calc.), i.e., starting from the 12mer, the mass reference list shifts to refer the ^13^C_1_ ionic compositions, e.g., [^13^C_1_C_87_H_95_N_12_]^+^, *m/z* 1320.78308 (calc.) as reference ions. Then, starting with the 25mer, the list shifts to refer the ^13^C_2_ ionic compositions, e.g., [^13^C_2_C_177_H_186_N_25_]^+^, *m/z* 2687.53847 (calc.). Finally, from the 39mer, [^13^C_3_C_274_H_284_N_39_]^+^, *m/z* 4159.35170, to the 48mer, [^13^C_3_C_336_H_347_N_48_]^+^, *m/z* 5104.87234 (calc.), the list refers to the ^13^C_3_ ionic compositions.

**Table 1.** Compositions and calculated *m/z* values of P2VP [M + H]^+^ ions as used in mass reference lists for calibration.

	Calculated *m/z* of [M + H]^+^				
Ionic composition of 2mer to 48mer	Monoisotopic	^13^C_1_-Ion	^13^C_2_-Ion	^13^C_3_-Ion	Average mass
[C_4_H_9_(C_7_H_7_N)_2_H + H]^+^	269.20123				
[C_4_H_9_(C_7_H_7_N)_3_H + H]^+^	374.25907				
[C_4_H_9_(C_7_H_7_N)_4_H + H]^+^	479.31692				
[C_4_H_9_(C_7_H_7_N)_5_H + H]^+^	584.37477				
[C_4_H_9_(C_7_H_7_N)_6_H + H]^+^	689.43262				
[C_4_H_9_(C_7_H_7_N)_7_H + H]^+^	794.49047				795.092
[C_4_H_9_(C_7_H_7_N)_8_H + H]^+^	899.54832				900.230
[C_4_H_9_(C_7_H_7_N)_9_H + H]^+^	1004.60617				1005.367
[C_4_H_9_(C_7_H_7_N)_10_H + H]^+^	1109.66402				1110.505
[C_4_H_9_(C_7_H_7_N)_11_H + H]^+^	1214.72187				1215.642
[C_4_H_9_(C_7_H_7_N)_12_H + H]^+^	1319.77972	1320.78302			1320.780
[C_4_H_9_(C_7_H_7_N)_13_H + H]^+^	1424.83756	1425.84086			1425.917
[C_4_H_9_(C_7_H_7_N)_14_H + H]^+^	1529.89541	1530.89871			1531.054
[C_4_H_9_(C_7_H_7_N)_15_H + H]^+^	1634.95326	1635.95656			1636.192
[C_4_H_9_(C_7_H_7_N)_16_H + H]^+^	1740.01111	1741.01441			1741.329
[C_4_H_9_(C_7_H_7_N)_17_H + H]^+^	1845.06896	1846.07226			1846.467
[C_4_H_9_(C_7_H_7_N)_18_H + H]^+^	1950.12681	1951.13011			1951.604
[C_4_H_9_(C_7_H_7_N)_19_H + H]^+^	2055.18466	2056.18796			2056.742
[C_4_H_9_(C_7_H_7_N)_20_H + H]^+^	2160.24251	2161.24581			2161.879
[C_4_H_9_(C_7_H_7_N)_21_H + H]^+^	2265.30036	2266.30366			2267.017
[C_4_H_9_(C_7_H_7_N)_22_H + H]^+^	2370.35821	2371.36151			2372.154
[C_4_H_9_(C_7_H_7_N)_23_H + H]^+^	2475.41605	2476.41935			2477.291
[C_4_H_9_(C_7_H_7_N)_24_H + H]^+^		2581.47720			2582.429
[C_4_H_9_(C_7_H_7_N)_25_H + H]^+^		2686.53505	2687.53846		2687.566
[C_4_H_9_(C_7_H_7_N)_26_H + H]^+^		2791.59290	2792.59631		2792.704
[C_4_H_9_(C_7_H_7_N)_27_H + H]^+^		2896.65075	2897.65416		2897.841
[C_4_H_9_(C_7_H_7_N)_28_H + H]^+^		3001.70860	3002.71201		3002.979
[C_4_H_9_(C_7_H_7_N)_29_H + H]^+^		3106.76645	3107.76986		3108.116
[C_4_H_9_(C_7_H_7_N)_30_H + H]^+^		3211.82430	3212.82771		3213.253
[C_4_H_9_(C_7_H_7_N)_31_H + H]^+^		3316.88215	3317.88556		3318.391
[C_4_H_9_(C_7_H_7_N)_32_H + H]^+^		3421.94000	3422.94341		3423.528
[C_4_H_9_(C_7_H_7_N)_33_H + H]^+^		3526.99784	3528.00125		3528.666
[C_4_H_9_(C_7_H_7_N)_34_H + H]^+^			3633.05910		3633.803
[C_4_H_9_(C_7_H_7_N)_35_H + H]^+^			3738.11695		3738.941
[C_4_H_9_(C_7_H_7_N)_36_H + H]^+^			3843.17480		3844.078
[C_4_H_9_(C_7_H_7_N)_37_H + H]^+^			3948.23265	3949.23601	3949.216
[C_4_H_9_(C_7_H_7_N)_38_H + H]^+^			4053.29050	4054.29385	4054.353
[C_4_H_9_(C_7_H_7_N)_39_H + H]^+^			4158.34835	4159.35170	4159.490
[C_4_H_9_(C_7_H_7_N)_40_H + H]^+^			4263.40620	4264.40955	4264.628
[C_4_H_9_(C_7_H_7_N)_41_H + H]^+^			4368.46405	4369.46740	4369.765
[C_4_H_9_(C_7_H_7_N)_42_H + H]^+^			4473.52190	4474.52525	4474.903
[C_4_H_9_(C_7_H_7_N)_43_H + H]^+^				4579.58310	4580.040
[C_4_H_9_(C_7_H_7_N)_44_H + H]^+^				4684.64095	4685.178
[C_4_H_9_(C_7_H_7_N)_45_H + H]^+^				4789.69880	4790.315
[C_4_H_9_(C_7_H_7_N)_46_H + H]^+^				4894.75665	4895.453
[C_4_H_9_(C_7_H_7_N)_47_H + H]^+^				4999.81450	5000.590
[C_4_H_9_(C_7_H_7_N)_48_H + H]^+^				5104.87234	5105.727

To deal with the detection of envelopes covering the isotopic pattern in case of higher-mass ions in linear mode TOF-MS, Table 1 also provides a column with [M + H]^+^ ion average masses.

### Reflector mode TOF-MS

Axial TOF instruments can still be considered the (gold) standard in MALDI-MS, and thus, the data obtained by reflector mode MALDI-TOF instrumentation shall be discussed first The present instrument, typically providing a mass resolving power of 15,000 at *m/z* 1000, easily delivered isotopic resolution over the entire range covered by the P2VPs. Based on external calibration, the achievable mass accuracy depended on the spatial distance between sample spot and reference spot and on the temporal gap between measuring these two. Provided the reference was placed on an adjacent spot and both the sample and the mass calibrant spectrum were acquired within about one minute, the mass accuracy was in the 3–10 ppm range. Polyethylene glycols (PEGs) provide well-defined series of [M + Na]^+^ and/or [M + K]^+^ ions in MALDI-MS.^24,25^ Here, PEG 600, PEG 1000, and PEG 1500 were prepared in DCTB matrix. The data obtained by alternating acquisition of spectra of P2VP, performing mass calibration and applying this to the next spectrum to be acquired of PEG 1000 is presented in Table 2. While both [M + Na]^+^ and [M + K]^+^ ions of PEG 1000 were formed, for clarity, only the more abundant [M + K]^+^ ions were considered here. Analogous results were also obtained with PEG 600 and PEG 1500 as long as the *m/z* range of the PEGs was covered by reference peaks of a suitable P2VP oligomer. Overall, the level of mass accuracy required for formula determination was normally not achieved by external mass calibration. Nonetheless, these calibrations were stable at ±0.2 u for weeks, and could thus be used as long as accurate mass was not required.

**Table 2.** Mass accuracy by reflector mode MALDI-TOF-MS based on external calibration. Data of PEG 1000 [M + K]^+^ ions, five repetitions, standard deviation of *m/z* in ppm, average error Δ(*m/z*) in mu. Conservatively, the accuracy level is at 10 ppm.

Formula	Calc. *m/*z	Exp. *m/z*	Exp. *m/z*	Exp. *m/z*	Exp. *m/z*	Exp. *m/z*	Avg. Exp. *m/z*	Std. Dev.
[C_36_H_74_O_19_K]^+^	849.4456	849.4424	849.4408	849.4365	849.4375	849.4386	849.4392	2.8
Δ(*m/z*)		0.0032	0.0048	0.0091	0.0081	0.0070	0.0064	7.6
[C_38_H_78_O_20_K]^+^	893.4718	893.4699	893.4682	893.4645	893.4655	893.4658	893.4668	2.5
Δ(*m/z*)		0.0019	0.0036	0.0073	0.0063	0.0060	0.0050	5.6
[C_40_H_82_O_21_K]^+^	937.4980	937.4958	937.4921	937.4897	937.4906	937.4912	937.4919	2.5
Δ(*m/z*)		0.0022	0.0059	0.0083	0.0074	0.0068	0.0061	6.5
[C_42_H_86_O_22_K]^+^	981.5242	981.5241	981.5225	981.5191	981.5196	981.5222	981.5215	2.1
Δ(*m/z*)		0.0001	0.0017	0.0051	0.0046	0.0020	0.0027	2.8
[C_44_H_90_O_23_K]^+^	1025.5504	1025.5503	1025.5478	1025.5441	1025.5451	1025.5469	1025.5468	2.4
Δ(*m/z*)		0.0001	0.0026	0.0063	0.0053	0.0035	0.0036	3.5
[C_46_H_94_O_24_K]^+^	1069.5767	1069.5788	1069.5765	1069.5719	1069.5739	1069.5764	1069.5755	2.5
Δ(*m/z*)		−0.0021	0.0002	0.0048	0.0028	0.0003	0.0012	1.1
[C_48_H_98_O_25_K]^+^	1113.6029	1113.6077	1113.6063	1113.6003	1113.6035	1113.6064	1113.6048	2.7
Δ(*m/z*)		−0.0048	−0.0034	0.0026	−0.0006	−0.0035	−0.0020	−1.8
[C_50_H_102_O_26_K]^+^	1157.6291	1157.6362	1157.6351	1157.6280	1157.6323	1157.6355	1157.6334	2.9
Δ(*m/z*)		−0.0071	−0.0060	0.0011	−0.0032	−0.0064	−0.0043	−3.7
[C_52_H_106_O_27_K]^+^	1201.6553	1201.6641	1201.6612	1201.6550	1201.6598	1201.6630	1201.6606	3.0
Δ(*m/z*)		−0.0088	−0.0059	0.0003	−0.0045	−0.0077	−0.0053	−4.4
[C_54_H_110_O_28_K]^+^	1245.6815	1245.6892	1245.6875	1245.6781	1245.6885	1245.6934	1245.6873	4.5
Δ(*m/z*)		−0.0077	−0.0060	0.0034	−0.0070	−0.0119	−0.0058	−4.7
[C_56_H_114_O_29_K]^+^	1289.7077	1289.7195	1289.7126	1289.7068	1289.7149	1289.7167	1289.7141	3.7
Δ(*m/z*)		−0.0118	−0.0049	0.0009	−0.0072	−0.0090	−0.0064	−4.9
[C_58_H_118_O_30_K]^+^	1333.7340	1333.7506	1333.7447	1333.7348	1333.7470	1333.7509	1333.7456	4.9
Δ(*m/z*)		−0.0166	−0.0107	−0.0008	−0.0130	−0.0169	−0.0116	−8.7
[C_60_H_122_O_31_K]^+^	1377.7602	1377.7706	1377.7672	1377.7533	1377.7625	1377.7730	1377.7653	5.7
Δ(*m/z*)		−0.0104	−0.0070	0.0069	−0.0023	−0.0128	−0.0052	−3.7

**Figure 3.**
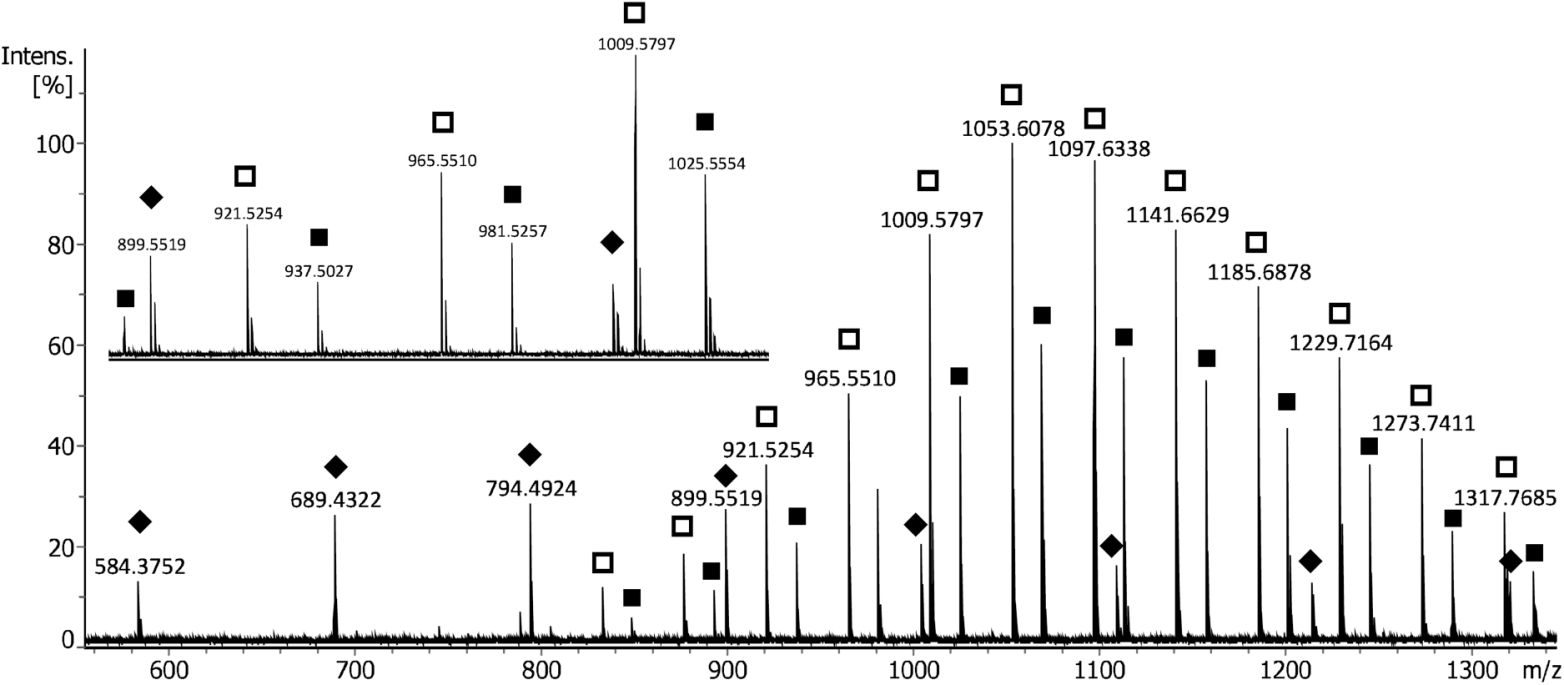
Positive-ion reflector mode MALDI-TOF spectrum of PVP-670 admixed to PEG 1000 in DCTB matrix. [PVP + H]^+^ ions are marked with diamonds, [PEG + Na]^+^ ions with empty squares, and [PEG + K]^+^ ions with filled squares. The insert shows that the gap between reference peaks is wide enough to encompass several analyte ion signals. However, there is some interference of calibrant and analyte with an isotopic peak of the [PEG + Na]^+^ ion at *m/z* 1317.7685.

When used as internal mass calibrant, the P2VPs were quite versatile. To avoid suppression of the analyte of interest one had to carefully adjust the amount of P2VP relative to the analyte to achieve an intensity ratio in the range of about 1 : 3 to 3 : 1. The positive-ion reflector mode MALDI-TOF spectrum of PVP-670 admixed to PEG 1000 exemplifies this approach. The spectrum shows [PVP + H]^+^ ions, [PEG + Na]^+^ ions, and [PEG + K]^+^ ions (Figure 3). Normally, the spacing of Δ(*m/z*) = 105.0578 between reference peaks is wide enough to encompass several analyte ion signals. However, there is some interference of calibrant and analyte could occur. In this particular spectrum there is an overlap with an isotopic peak of the [PEG + Na]^+^ ion at *m/z* 1317.7685. Proper operation provided, this technique generally delivered mass accuracies of 2–5 ppm (Table 3). Again, analogous results were also obtained with PEG 600 and PEG 1500 as long as the *m/z* range of the PEGs was covered by reference peaks of a suitable P2VP. Nonetheless, admixing the calibrant at a ratio that yielded well-defined reference peaks while not suppressing the analyte ions often required the preparation of several spots on the MALDI target. Further, this procedure is restricted to analytes tolerant to the basic P2VP calibrant.

**Table 3.** Mass accuracy by reflector mode MALDI-TOF-MS based on internal calibration. Data of PEG 1000 [M + Na]^+^ ions, five repetitions, standard deviation of *m/z* in ppm, average error Δ(*m/z*) in mu.

Formula	Calc. *m/z*	Exp. *m/z*	Exp. *m/z*	Exp*. m/z*	Exp. *m/z*	Exp. *m/z*	Avg. Exp. *m/z*	Std. Dev.
[C_36_H_74_O_19_Na]^+^	833.4717	833.4715	833.4700	833.4698	833.4700	833.4682	833.4699	1.4
Δ(*m/z*)		0.0002	0.0017	0.0019	0.0017	0.0035	0.0018	2.1
[C_38_H_78_O_20_Na]^+^	877.4979	877.4987	877.5000	877.4997	877.4979	877.4985	877.4990	1.0
Δ(*m/z*)		−0.0008	−0.0021	−0.0018	0.0000	−0.0006	−0.0011	−1.2
[C_40_H_82_O_21_Na]^+^	921.5241	921.5251	921.5261	921.5222	921.5232	921.5216	921.5236	2.1
Δ(*m/z*)		−0.0010	−0.0020	0.0019	0.0009	0.0025	0.0004	0.5
[C_42_H_86_O_22_Na]^+^	965.5503	965.5483	965.5483	965.5492	965.5464	965.5476	965.5480	1.1
Δ(*m/z*)		0.0020	0.0020	0.0011	0.0039	0.0027	0.0023	2.4
[C_44_H_90_O_23_Na]^+^	1009.5765	1009.5766	1009.5766	1009.5781	1009.5790	1009.5780	1009.5777	1.0
Δ(*m/z*)		−0.0001	−0.0001	−0.0016	−0.0025	−0.0015	−0.0011	−1.1
[C_46_H_94_O_24_Na]^+^	1053.6027	1053.6007	1053.6029	1053.6043	1053.6035	1053.6052	1053.6033	1.6
Δ(*m/z*)		0.0020	−0.0002	−0.0016	−0.0008	−0.0025	−0.0006	−0.6
[C_48_H_98_O_25_Na]^+^	1097.6289	1097.6276	1097.6289	1097.6285	1097.6307	1097.6308	1097.6293	1.3
Δ(*m/z*)		0.0013	0.0000	0.0004	−0.0018	−0.0019	−0.0004	−0.3
[C_50_H_102_O_26_Na]^+^	1141.6552	1141.6534	1141.6534	1141.6566	1141.6567	1141.6601	1141.6560	2.4
Δ(*m/z*)		0.0018	0.0018	−0.0014	−0.0015	−0.0049	−0.0009	−0.8
[C_52_H_106_O_27_Na]^+^	1185.6814	1185.6789	1185.6789	1185.6796	1185.6828	1185.6839	1185.6808	2.0
Δ(*m/z*)		0.0025	0.0025	0.0018	−0.0014	−0.0025	0.0005	0.5
[C_54_H_110_O_28_Na]^+^	1229.7076	1229.7074	1229.7080	1229.7072	1229.7123	1229.7116	1229.7093	2.0
Δ(*m/z*)		0.0002	−0.0004	0.0004	−0.0047	−0.0040	−0.0017	−1.4
[C_56_H_114_O_29_Na]^+^	1273.7338	1273.7307	1273.7277	1273.7337	1273.7353	1273.7390	1273.7333	3.4
Δ(*m/z*)		0.0031	0.0061	0.0001	−0.0015	−0.0052	0.0005	0.4
[C_58_H_118_O_30_Na]^+^	1317.7600	1317.7609	1317.7539	1317.7612	1317.7642	1317.7686	1317.7618	4.1
Δ(*m/z*)		−0.0009	0.0061	−0.0012	−0.0042	−0.0086	−0.0017	−1.3
[C_60_H_122_O_31_Na]^+^	1361.7862	1361.7837	1361.7911	1361.7796	1361.7826	1361.7866	1361.7847	3.2
Δ(*m/z*)		0.0025	−0.0049	0.0066	0.0036	−0.0004	0.0015	1.1

### Linear mode TOF-MS

**Figure 4.**
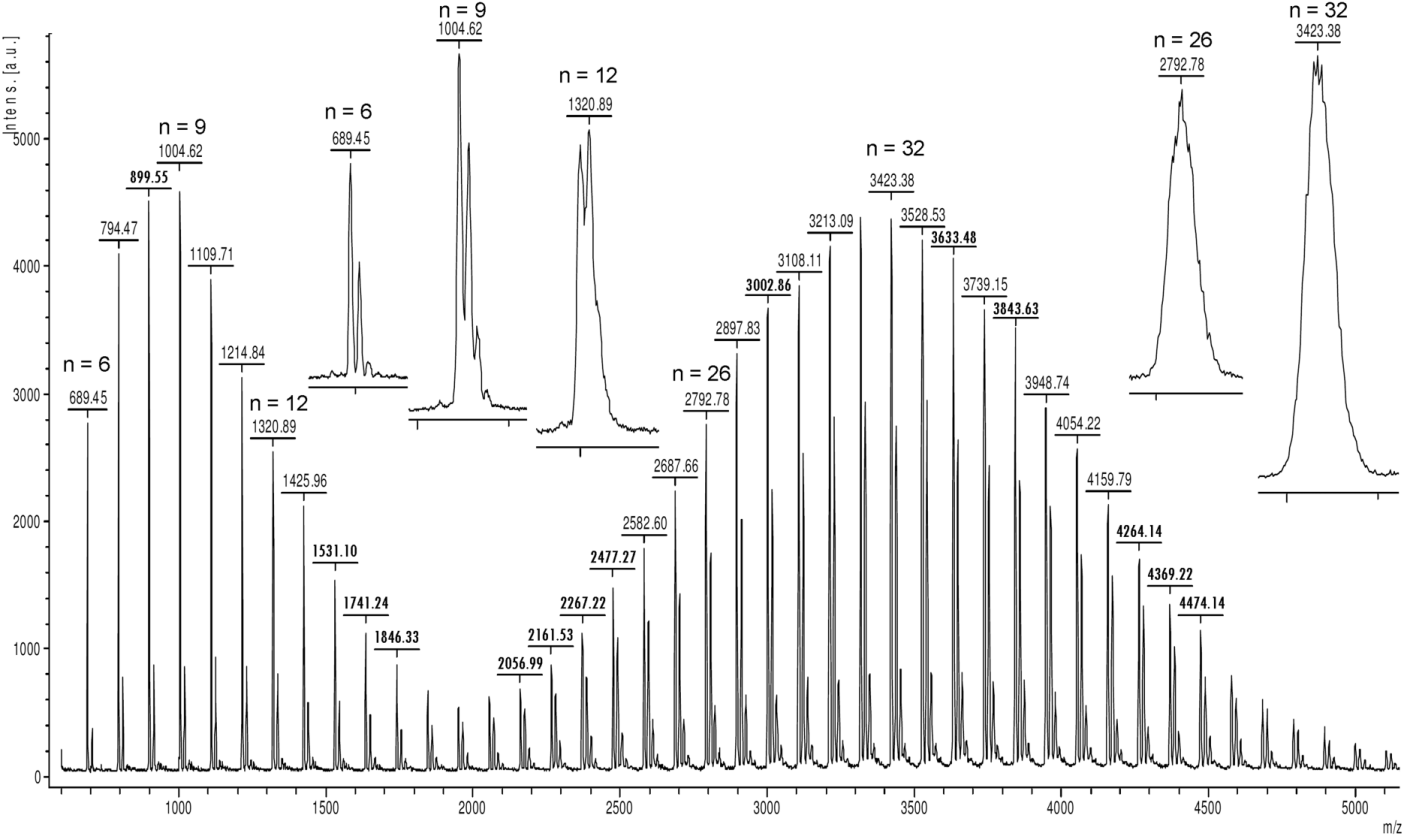
Positive-ion linear mode MALDI spectrum of a mixture of PVP-670 plus PVP-2.1k (1 : 3) in DCTB. The inserts show expanded views of signals corresponding to [M + H]^+^ ions across the *m/z* range exemplifying the transition from isotopic resolution (*n* = 6 and 9) over an intermediate zone (*n* = 12) to the detection of envelopes over the isotopic distribution (*n* = 26 and 32).

Using the TOF analyzer in linear mode introduces a limitation in mass resolving power. Thus, the positive-ion linear mode MALDI spectrum of a mixture of PVP-670 plus PVP-2.1k (1 : 3) covering the *m/z* 600–5000 range shows a transition from isotopic resolution over an intermediate zone to the detection of envelopes over the entire isotopic distribution (Figure 4). While the instrument used still delivered good isotopic separation up to the 10mer, the signals above about *m/z* 1200 showed a coalescence that finally resulted in isotopically unresolved envelopes for the 20mer and larger ions. This gradual loss of isotopic resolution is exemplified by the inserts of Figure 4 showing expanded views of signals corresponding to [M + H]^+^ ions of the resolved peaks related to the 6mer and 9mer, to skewed peaks of the 12mer, and finally to evenly shaped envelopes as in case of the 26mer or 32mer.

For mass calibration, this dictates that depending on the actual resolving power, monoisotopic *m/z* values need to be employed in the lower portion of the *m/z* range while average masses are to be used at higher *m/z*. With the particular instrument, sufficient resolution typically allowed for the assignment of the monoisotopic *m/z* values up to the 10mer (*m/z* 1109.66) whereas the transition from isotopic resolution to a sufficiently uniform envelope over the isotopic peaks caused problems in this regard as the peak position neither reflects the neat monoisotopic nor the correct average mass of the ion. Signals above the 22mer (*m/z* 2372.12) reflected quite clean envelopes of the isotopic distribution and could confidently be assigned to average mass values. In practice, calibration of the wide *m/z* range in linear mode was realized by selecting and including all monoisotopic ions up the 11mer, then omitting poorly defined peaks of the intermediate region, and finally assigning the evenly shaped envelope signals to average *m/z* values. The calibration was then performed using the “cubic enhanced” algorithm provided by the instrument software.

The need to interpolate the mid-range combined with limited resolution in the low-mass range resulted in a comparatively low mass accuracy as demonstrated on oligosaccharide [M + Na]^+^ ions (Table 4). The oligosaccharides were extracted from gummy bears and admixed to DHB matrix.^37,38^ While ions starting from [C_84_H_142_O_71_Na]^+^, *m/z* 2310.976 (calc.) and up to [C_114_H_192_O_96_Na]^+^, *m/z* 3121.680 (calc.) appeared at a Δ(*m/z*) of less than 0.15 u, the ions below tended to deviate from the calculated value by about 0.25–0.40 u. In case of linear mode TOF-MS, the established [Cs*_n_* I*_n_*_–1_]^+^ cluster ion calibration^17–19^ admittedly provided much better accuracy, because there, isotopic resolution is not an issue.

**Table 4.** Mass accuracy by linear mode MALDI-TOF-MS based on external calibration. Data of oligosaccharide [M + Na]^+^ ions, five repetitions, standard deviation of *m/z* in ppm, average error Δ(*m/z*) in mu.

Formula	Calc. *m/z*	Exp. *m/z*	Exp. *m/z*	Exp. *m/z*	Exp. *m/z*	Exp. *m/z*	Avg. Exp. *m/z*	Std. Dev.
[C_24_H_42_O_21_Na]^+^	689.568	689.251	689.255	689.253	689.252	689.237	689.250	10.4
Δ(*m/z*)		0.317	0.313	0.315	0.316	0.331	0.318	461.8
[C_30_H_52_O_26_Na]^+^	851.709	851.335	851.338	851.335	851.336	851.331	851.335	3.0
Δ(*m/z*)		0.374	0.371	0.374	0.373	0.378	0.374	439.0
[C_36_H_62_O_31_Na]^+^	1013.849	1013.467	1013.473	1013.470	1013.472	1013.470	1013.470	2.3
Δ(*m/z*)		0.383	0.377	0.380	0.378	0.380	0.379	374.1
[C_42_H_72_O_36_Na]^+^	1175.990	1175.600	1175.610	1175.606	1175.612	1175.597	1175.605	5.4
Δ(*m/z*)		0.390	0.380	0.384	0.378	0.393	0.385	327.7
[C_48_H_82_O_41_Na]^+^	1338.131	1337.759	1337.761	1337.758	1337.765	1337.748	1337.758	4.7
Δ(*m/z*)		0.372	0.370	0.373	0.366	0.383	0.373	278.8
[C_54_H_92_O_46_Na]^+^	1500.272	1499.981	1499.882	1499.885	1499.896	1499.888	1499.906	28.0
Δ(*m/z*)		0.291	0.390	0.387	0.376	0.384	0.366	243.7
[C_60_H_102_O_51_Na]^+^	1662.413	1661.995	1662.018	1662.036	1662.007	1661.997	1662.011	10.2
Δ(*m/z*)		0.418	0.395	0.377	0.406	0.416	0.402	241.9
[C_66_H_112_O_56_Na]^+^	1824.554	1824.873	1824.883	1824.873	1824.884	1824.872	1824.877	3.3
Δ(*m/z*)		−0.319	−0.329	−0.319	−0.330	−0.318	−0.323	−177.3
[C_72_H_122_O_61_Na]^+^	1986.694	1986.973	1987.000	1986.990	1986.992	1986.959	1986.983	8.3
Δ(*m/z*)		−0.279	−0.306	−0.296	−0.298	−0.265	−0.288	−145.2
[C_78_H_132_O_66_Na]^+^	2148.835	2149.038	2149.067	2149.048	2149.111	2149.038	2149.060	14.3
Δ(*m/z*)		−0.203	−0.232	−0.213	−0.276	−0.203	−0.225	−104.8
[C_84_H_142_O_71_Na]^+^	2310.976	2311.091	2311.097	2311.121	2311.145	2311.104	2311.112	9.4
Δ(*m/z*)		−0.115	−0.121	−0.145	−0.169	−0.128	−0.136	−58.7
[C_90_H_152_O_76_Na]^+^	2473.117	2473.152	2473.208	2473.152	2473.136	2473.149	2473.159	11.3
Δ(*m/z*)		−0.035	−0.091	−0.035	−0.019	−0.032	−0.043	−17.3
[C_96_H1_62_O_81_Na]^+^	2635.258	2635.218	2635.234	2635.255	2635.257	2635.294	2635.252	10.9
Δ(*m/z*)		0.040	0.024	0.003	0.001	−0.036	0.006	2.2
[C_102_H_172_O_86_Na]^+^	2797.398	2797.244	2797.214	2797.257	2797.185	2797.284	2797.237	13.7
Δ(*m/z*)		0.154	0.184	0.141	0.213	0.114	0.162	57.7
[C_108_H_182_O_91_Na]^+^	2959.539	2959.498	2959.228	2959.255	2959.273	2959.444	2959.340	41.4
Δ(*m/z*)		0.041	0.311	0.284	0.266	0.095	0.200	67.4
[C_114_H_192_O_96_Na]^+^	3121.680	3121.935	3121.293	3121.932	3121.334	3121.538	3121.606	100.2
Δ(*m/z*)		−0.255	0.387	−0.252	0.346	0.142	0.074	23.5

### IM-Q-TOF-MS and FT-ICR-MS

The Bruker timsTOF flex ion mobility-quadrupole-time-of-flight (IM-Q-TOF) mass spectrometer and the Bruker Apex-Qe Fourier transform ion cyclotron resonance (FT-ICR) mass spectrometer not only shared the capability to deliver more than sufficient resolving power (*R* ≥ 40.000) and to deliver long-term stability of their mass calibration, they even shared the same mass reference file. As mentioned above (Table 1), isotopolog ions yield the most intensive signal within isotopic patterns of larger calibrant ions, and thus, the common mass reference list for these instruments was based on the respective ions. In particular with the IM-Q-TOF instrument the peak distribution of positive-ion spectra of a mixture composed of PVP-067 and PVP-2.1k as well as of each of the P2VPs alone exhibited essentially the same appearance as observed in reflector TOF-MS (Fig. S13). Either instrument was able to automatically pick the correct reference peak across the entire range and to perform mass calibration (Fig. S14).

**Figure 5.**
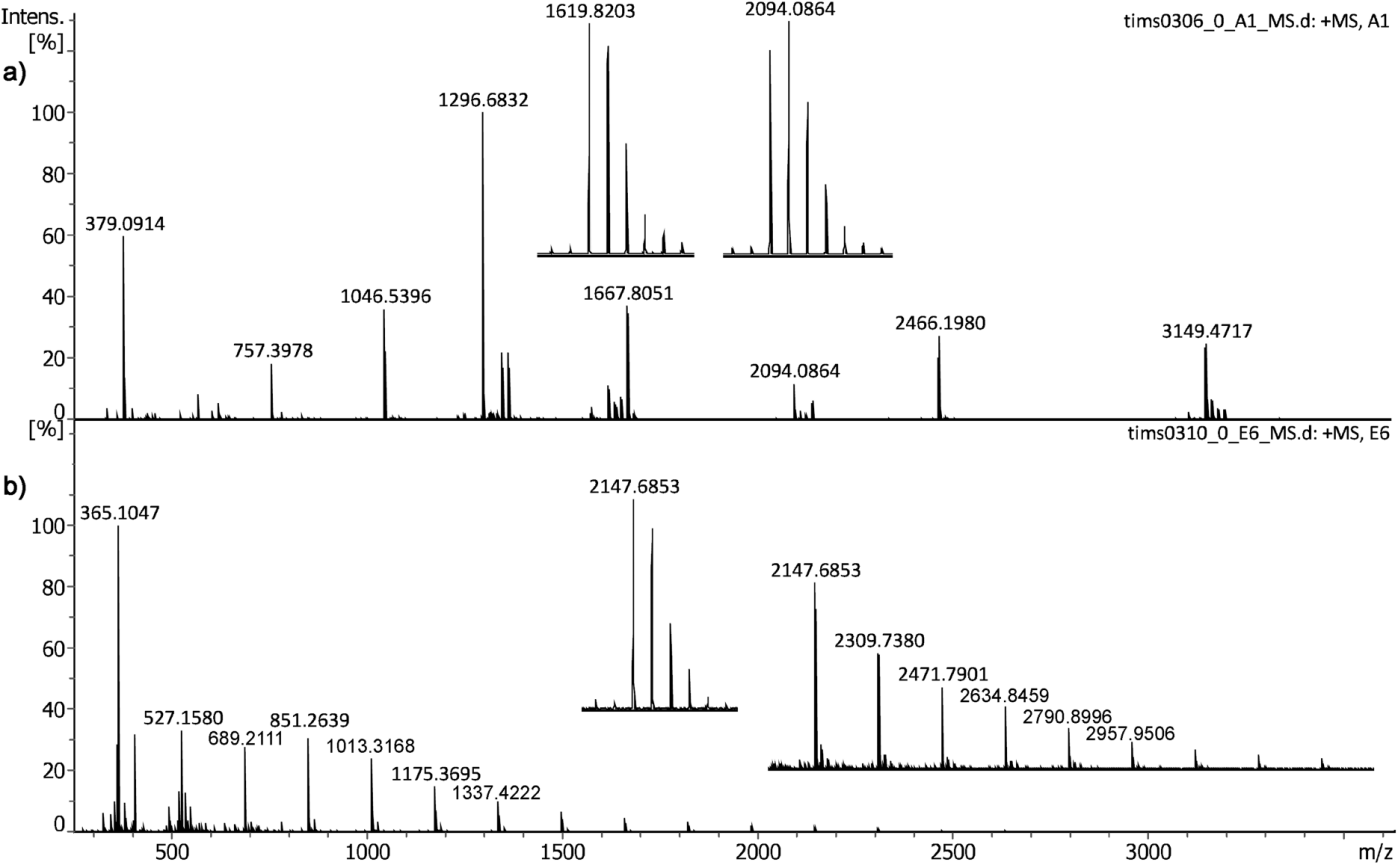
Positive-ion MALDI spectra of (a) Bruker Peptide Mix II in CHCA matrix and (b) oligosaccharides from gummy bears in DHB matrix as obtained using the Bruker timsTOF flex instrument. The spectra are part of the dataset used to compile Tables 5 and 6. In (a), the inserts show expanded views of the [M + H]^+^ ions of bombesin, *m/z* 1619.8223 (calc.) and of ACTH clip 1–17, *m/z* 2093.0862 (calc.). In (b) the inserts show expanded views of the *m/z* range above 2000 and of the [C_78_H_132_O_66_Na]^+^ ion, *m/z* 2147.6865 (calc.). Labels of ions beyond *m/z* 2000 may refer to the first isotopic peak.

As in linear TOF-MS, gummy bears were used as a source of oligosaccharides and [M + Na]^+^ ions appeared as prevalent species in the spectra. The signals observed ranged from the disaccharide sucrose, [C_12_H_22_O_11_Na]^+^, *m/z* 365.1054 (calc.) to the 17mer oligosaccharide [C_102_H_172_O_86_Na]^+^, *m/z* 2795.8978 (calc.). The IM-Q-TOF instrument delivered data with clearly better than 2 ppm mass accuracy (Table 5). Additionally, spectra of [peptide + H]^+^ ions as delivered by the Bruker Standard Peptide Mix II were acquired from CHCA matrix. Again, all ions from the [(CHCA)_2_ + H]^+^ matrix cluster ion, *m/z* 379.0924 (calc.) to the largest peptide of the mixture, somatostatin 28, *m/z* 3147.4710 (calc.) were well within 2 ppm error (Table 6). As examples, one spectrum of each series as obtained using the Bruker timsTOF flex instrument is depicted in Figure 5.

**Table 5.** Mass accuracy by IM-Q-TOF-MS based on external calibration. Data of oligosaccharide [M + Na]^+^ ions. Signals were observed from the disaccharide sucrose, [C_12_H_22_O_11_Na]^+^, *m/z* 365.1054 (calc.) to the 17mer oligosaccharide [C_102_H_172_O_86_Na]^+^, *m/z* 2795.8978 (calc.). Data of five repetitions, standard deviation of *m/z* in ppm, average error Δ(*m/z*) in mu.

Formula	Calc. *m/z*	Exp. *m/z*	Exp. *m/z*	Exp. *m/z*	Exp. *m/z*	Exp. *m/z*	Avg. Exp. *m/z*	Std. Dev.
[C_12_H_22_O_11_Na]^+^	365.1054	365.1048	365.1047	365.1048	365.1048	365.1050	365.1048	0.3
Δ(*m/z*)		0.0006	0.0007	0.0006	0.0006	0.0004	0.0006	1.7
[C_18_H_32_O_16_Na]^+^	527.1583	527.1581	527.1580	527.1581	527.1581	527.1583	527.1581	0.2
Δ(*m/z*)		0.0002	0.0003	0.0002	0.0002	0.0000	0.0001	0.3
[C_24_H_42_O_21_Na]^+^	689.2111	689.2113	689.2111	689.2113	689.2112	689.2115	689.2113	0.2
Δ(*m/z*)		−0.0002	0.0000	−0.0002	−0.0001	−0.0004	−0.0002	−0.3
[C_30_H_52_O_26_Na]^+^	851.2639	851.2640	851.2639	851.2640	851.2640	851.2643	851.2640	0.2
Δ(*m/z*)		−0.0001	0.0000	−0.0001	−0.0001	−0.0004	−0.0001	−0.2
[C_36_H_62_O_31_Na]^+^	1013.3167	1013.3168	1013.3168	1013.3168	1013.3169	1013.3174	1013.3169	0.3
Δ(*m/z*)		−0.0001	−0.0001	−0.0001	−0.0002	−0.0007	−0.0002	−0.2
[C_42_H_72_O_36_Na]^+^	1175.3695	1175.3697	1175.3695	1175.3697	1175.3697	1175.3702	1175.3698	0.2
Δ(*m/z*)		−0.0002	0.0000	−0.0002	−0.0002	−0.0007	−0.0002	−0.2
[C_48_H_82_O_41_Na]^+^	1337.4224	1337.4224	1337.4222	1337.4224	1337.4225	1337.4231	1337.4225	0.3
Δ(*m/z*)		0.0000	0.0002	0.0000	−0.0001	−0.0007	−0.0002	−0.1
[C_54_H_92_O_46_Na]^+^	1499.4752	1499.4751	1499.4750	1499.4752	1499.4754	1499.4758	1499.4753	0.2
Δ(*m/z*)		0.0001	0.0002	0.0000	−0.0002	−0.0006	−0.0001	−0.1
[C_60_H_102_O_51_Na]^+^	1661.5280	1661.5276	1661.5276	1661.5278	1661.5282	1661.5283	1661.5279	0.2
Δ(*m/z*)		0.0004	0.0004	0.0002	−0.0002	−0.0003	0.0001	0.1
[C_66_H_112_O_56_Na]^+^	1823.5808	1823.5803	1823.5802	1823.5807	1823.5805	1823.5815	1823.5806	0.3
Δ(*m/z*)		0.0005	0.0006	0.0001	0.0003	−0.0007	0.0002	0.1
[C_72_H_122_O_61_Na]^+^	1985.6337	1985.6329	1985.6331	1985.6319	1985.6329	1985.6343	1985.6330	0.4
Δ(*m/z*)		0.0008	0.0006	0.0018	0.0008	−0.0006	0.0006	0.3
[C_78_H_132_O_66_Na]^+^	2147.6865	2147.6861	2147.6853	2147.6848	2147.6858	2147.6864	2147.6857	0.3
Δ(*m/z*)		0.0004	0.0012	0.0017	0.0007	0.0001	0.0008	0.4
[C_84_H_142_O_71_Na]^+^	2309.7393	2309.7391	2309.7380	2309.7377	2309.7374	2309.7426	2309.7390	0.9
Δ(*m/z*)		0.0002	0.0013	0.0016	0.0019	−0.0033	0.0003	0.2
[C_90_H_152_O_76_Na]^+^	2471.7921	2471.7888	2471.7901	2471.7883	2471.7901	2471.7919	2471.7898	0.6
Δ(*m/z*)		0.0033	0.0020	0.0038	0.0020	0.0002	0.0023	0.9
[C_96_H_162_O_81_Na]^+^	2633.8450	2633.8435	2633.8428	2633.8436	2633.8455	2633.8425	2633.8436	0.4
Δ(*m/z*)		0.0015	0.0022	0.0014	−0.0005	0.0025	0.0014	0.5
[C_102_H_172_O_86_Na]^+^	2795.8978	2795.8964	2795.8942	2795.8935	2795.8982	2795.9014	2795.8967	1.1
Δ(*m/z*)		0.0014	0.0036	0.0043	−0.0004	−0.0036	0.0010	0.4

**Table 6.** Mass accuracy by IM-Q-TOF-MS based on external calibration. Data of [peptide + H]^+^ ions of the Bruker Standard Peptide Mix II in CHCA. Data of five repetitions, standard deviation of *m/z* in ppm, average error Δ(*m/z*) in mu.

Formula	Calc. *m/z*	Exp. *m/z*	Exp. *m/z*	Exp. *m/z*	Exp. *m/z*	Exp. *m/z*	Avg. Exp. *m/z*	Std. Dev.
[(C_10_H_7_NO_3_)_2_ + H]^+^	379.0924	379.0915	379.0917	379.0918	379.0914	379.0917	379.0916	0.4
Δ(*m/z*)		0.0009	0.0007	0.0006	0.0010	0.0007	0.0008	2.1
Bradykinin 1–7	757.3992	757.3986	757.3989	757.3992	757.3978	757.3988	757.3987	0.7
Δ(*m/z*)		0.0006	0.0003	0.0000	0.0014	0.0004	0.0005	0.7
Angiotensin II	1046.5418	1046.5411	1046.5417	1046.5420	1046.5396	1046.5415	1046.5412	0.9
Δ(*m/z*)		0.0007	0.0001	−0.0002	0.0022	0.0003	0.0006	0.6
Angiotensin I	1296.6848	1296.6838	1296.6843	1296.6852	1296.6832	1296.6849	1296.6843	0.6
Δ(*m/z*)		0.0010	0.0005	−0.0004	0.0016	−0.0001	0.0005	0.4
Substance P	1347.7354	1347.7343	1347.7347	1347.7353	1347.7353	1347.7348	1347.7349	0.3
Δ(*m/z*)		0.0011	0.0007	0.0001	0.0001	0.0006	0.0005	0.4
Bombesin	1619.8223	1619.8209	1619.8214	1619.8219	1619.8203	1619.8213	1619.8212	0.4
Δ(*m/z*)		0.0014	0.0009	0.0004	0.0020	0.0010	0.0011	0.7
ACTH clip 1–17	2093.0862	2093.0832	2093.0844	2093.0857	2093.0832	2093.0852	2093.0843	0.5
Δ(*m/z*)		0.0030	0.0018	0.0005	0.0030	0.0010	0.0019	0.9
ACTH clip 18–39	2465.1983	2465.1954	2465.1964	2465.1977	2465.1948	2465.1969	2465.1962	0.5
Δ(*m/z*)		0.0029	0.0019	0.0006	0.0035	0.0014	0.0021	0.8
Somatostatin 28	3147.4710	3147.4662	3147.4672	3147.4695	3147.4656	3147.4683	3147.4674	0.5
Δ(*m/z*)		0.0048	0.0038	0.0015	0.0054	0.0027	0.0036	1.2

Analogous results were obtained using the FT-ICR instrument (Table S3 and Fig. S15) the main difference being that the 14-year old instrument started to show its age. Thus, with the given instrument, MALDI-FT-ICR spectra were somewhat inferior to the IM-Q-TOF spectra, mostly in terms of signal-to-noise ratio, and as a result, also in mass accuracy. Nonetheless, either instrument worked flawlessly with the P2VP reference file.

## Conclusions

P2PVs are demonstrated as versatile reference compounds for external and internal mass calibration due to the formation of clean [M + H]^+^ ion series. P2VPs are compatible with CHCA, DHB, dithranol, and DCTB matrix and a wide range of analyte polarities ranging from ionic to nonpolar. In our laboratory DCTB proved most useful due its wide range of analyte acceptance and low laser fluence requirements.

P2VP-based mass calibration can be applied in MALDI-MS with a linear TOF analyzer, however, the mid-range drop in mass accuracy renders it inferior to calibration compounds delivering monoisotopic ions.

The mass accuracy based on external calibration of the reflector MALDI-TOF instrument was in the range of 3–10 ppm, and thus, generally not sufficient for reliable formula determination based on accurate mass. However, using P2VPs for internal mass calibration allowed to routinely achieve mass accuracies in the 2–5 ppm range for analytes of molecular mass between 300 u and 1500 u when using the reflector MALDI-TOF instrument with pulsed ion extraction. Notably higher levels of mass accuracy, i.e., 1–2 ppm, were achieved when higher-resolving analyzers like a modern IM-Q-TOF analyzer or an FT-ICR instrument were being used. Remarkably, IM-Q-TOF analyzer and FT-ICR instrument were able to provide this 1–2 ppm mass accuracy with external calibration all day long.

The reference data (as MS Excel sheet, as *.mcl files for linear and reflector mode TOF-MS in Bruker FlexControl 3.4 format, and as *.ref files for IM-Q-TOF Bruker timsControl and FT-ICR-MS for Bruker ApexControl, respectively) can be obtained from the author upon request

## Supplemental Material

sj-pdf-1-ems-10.1177_14690667211055701 - Supplemental material for Poly(2-vinylpyridine) as a reference compound for mass calibration in positive-ion matrix-assisted laser desorption/ionization-mass spectrometry on different instrumental platforms Click here for additional data file. Supplemental material, sj-pdf-1-ems-10.1177_14690667211055701 for Poly(2-vinylpyridine) as a reference compound for mass calibration in positive-ion matrix-assisted laser desorption/ionization-mass spectrometry on different instrumental platforms by Jürgen H Gross in European Journal of Mass Spectrometry
