# Supplementary material for: Poly(2-vinylpyridine) as a reference compound for mass calibration in positive-ion matrix-assisted laser desorption/ionization-mass spectrometry on different instrumental platforms
Source: Eur J Mass Spectrom (Chichester). 2021 Nov 5;27(5):191–204. doi: 10.1177/14690667211055701 (PMC8586192; doi:10.1177/14690667211055701)
Supplement: sj-pdf-1-ems-10.1177_14690667211055701 - Supplemental material for Poly(2-vinylpyridine) as a reference compound for mass calibration in positive-ion matrix-assisted laser desorption/ionization-mass spectrometry on different instrumental platforms [file sj-pdf-1-ems-10.1177_14690667211055701.pdf]

Poly(2-Vinylpyridine) as a Reference Compound for Mass Calibration in Positive-Ion Matrix-Assisted Laser Desorption/Ionization-Mass Spectrometry on Different Instrumental Platforms

Supplementary Data

Jürgen H. Gross

ORCID 0000-0003-0748-2535

Address

Dr. Jürgen H. Gross

Institute of Organic Chemistry

Heidelberg University

Im Neuenheimer Feld 270

69120 Heidelberg

Germany

email: juergen.gross@oci.uni-heidelberg.de

phone: +49/6221/54-8409

fax: +49/6221/54-4205

Table S1. Essential instrumental settings of the Bruker Autoflex MALDI-TOF instrument for ranges typically used in linear mode and reflector modes, respectively.

	**Linear mode** ***m/z*** **range**	**Reflector mode** ***m/z*** **range A**	**Reflector mode** ***m/z*** **range B**
m/z **Range**	500–5000	200–2000	440–5000
**Ion source 1 [kV]**	19.50	19.00	19.00
**Ion source 2 [kV]**	18.25	16.50	16.65
**Lens [kV]**	7.00	8.00	8.00
**Reflector [kV]**	-	21.00	21.00
**Reflector 2 [kV]**	-	9.45	9.45
**PIE Delay [ns]**	150	100	140
**Matrix suppression up to [Da]**	580	180	420
**Digitizer rate [GHz]**	1.25	5.00	5.00
**Smartbeam laser setting**	4_large	3_medium	3_medium
**Laser repetition rate [Hz]**	2000	2000	2000

Table S2. Essential instrumental settings of the Bruker timsTOF flex instrument in MALDI mode operation.

***m/z* Range**	250–5000
**Smartbeam laser setting**	Dried droplet
**Sample carrier**	Movement random, partial sample
**Laser repetition rate [Hz]**	10000
**Deflection plate [V]**	70
**MALDI plate offset [V]**	50
**Funnel 1 RF [Vpp]**	400
**Funnel 2 RF [Vpp]**	430
**Multipole RF [Vpp]**	480
**Quadrupole low mass [Da]**	250
**Collision cell RF [Vpp]**	1800
**Pre TOF transfer time [µs]**	130
**Pre pulse storage time [µs]**	12

Fig. S1. Positive-ion linear mode MALDI-TOF spectra of PVP-067, PVP-1k, and PVP-2.1k (*top* to *bottom*) in CHCA matrix at laser power set to 15 %, 15%, and 25 %, respectively. All spectra are dominated by $[M+H]^+$ ions.

General remark on peak labels: The peak picking algorithm generally puts the *m/z* label at the most abundant peak of the respective isotopic pattern, i.e., it refers to the monoisotopic ion only as long as this contributes the most intensive peak within the group. In case of linear mode spectra, there is a transition from labels at the monoisotopic peak to average *m/z* of the non-resolved envelope at higher *m/z*. Refer to tables for accurate assignments.

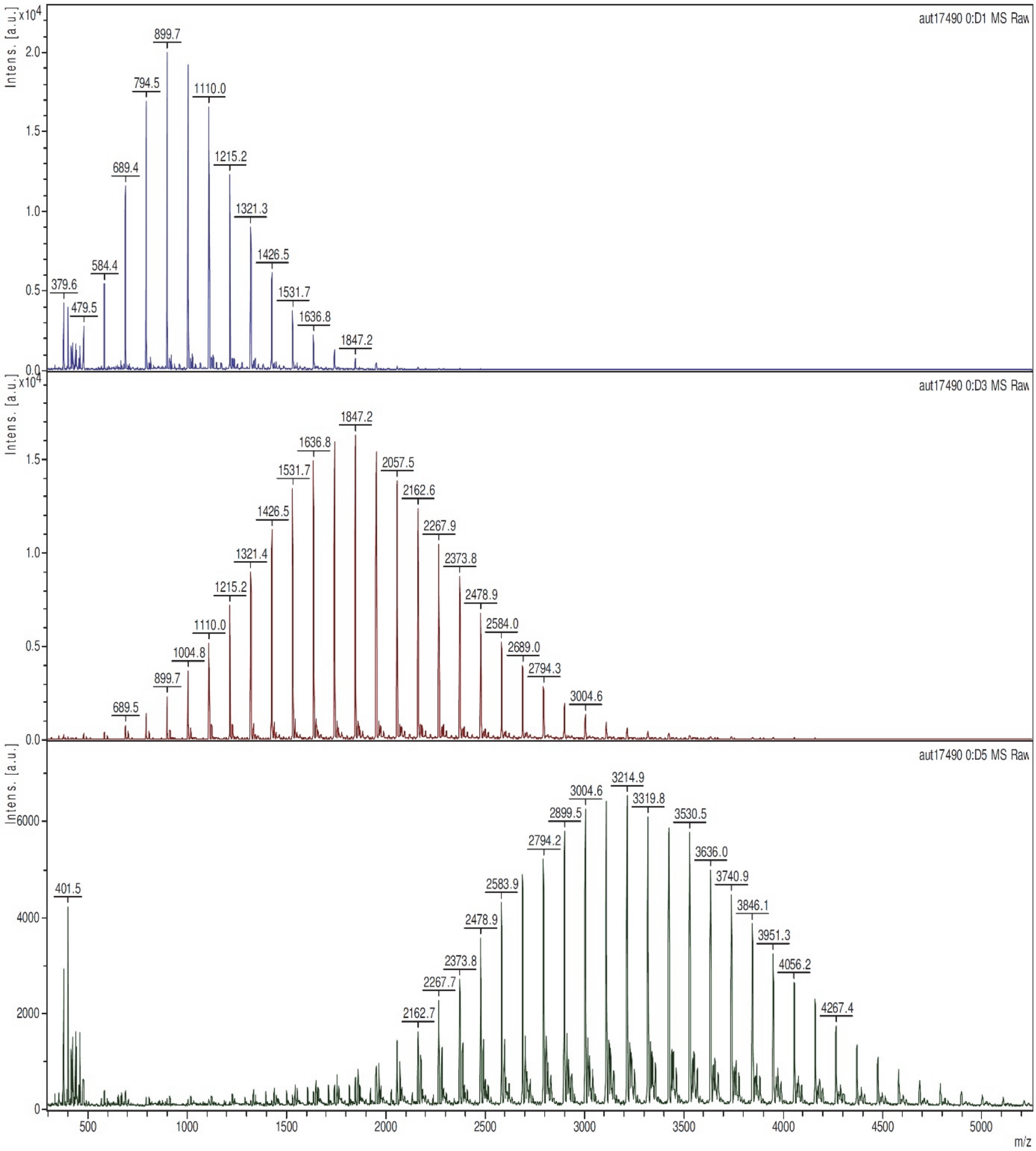

Fig. S2. Positive-ion reflector mode MALDI-TOF spectra of PVP-067, PVP-1k, and PVP-2.1k (*top* to *bottom*) in CHCA matrix . All spectra are dominated by $[M+H]^+$ ions.

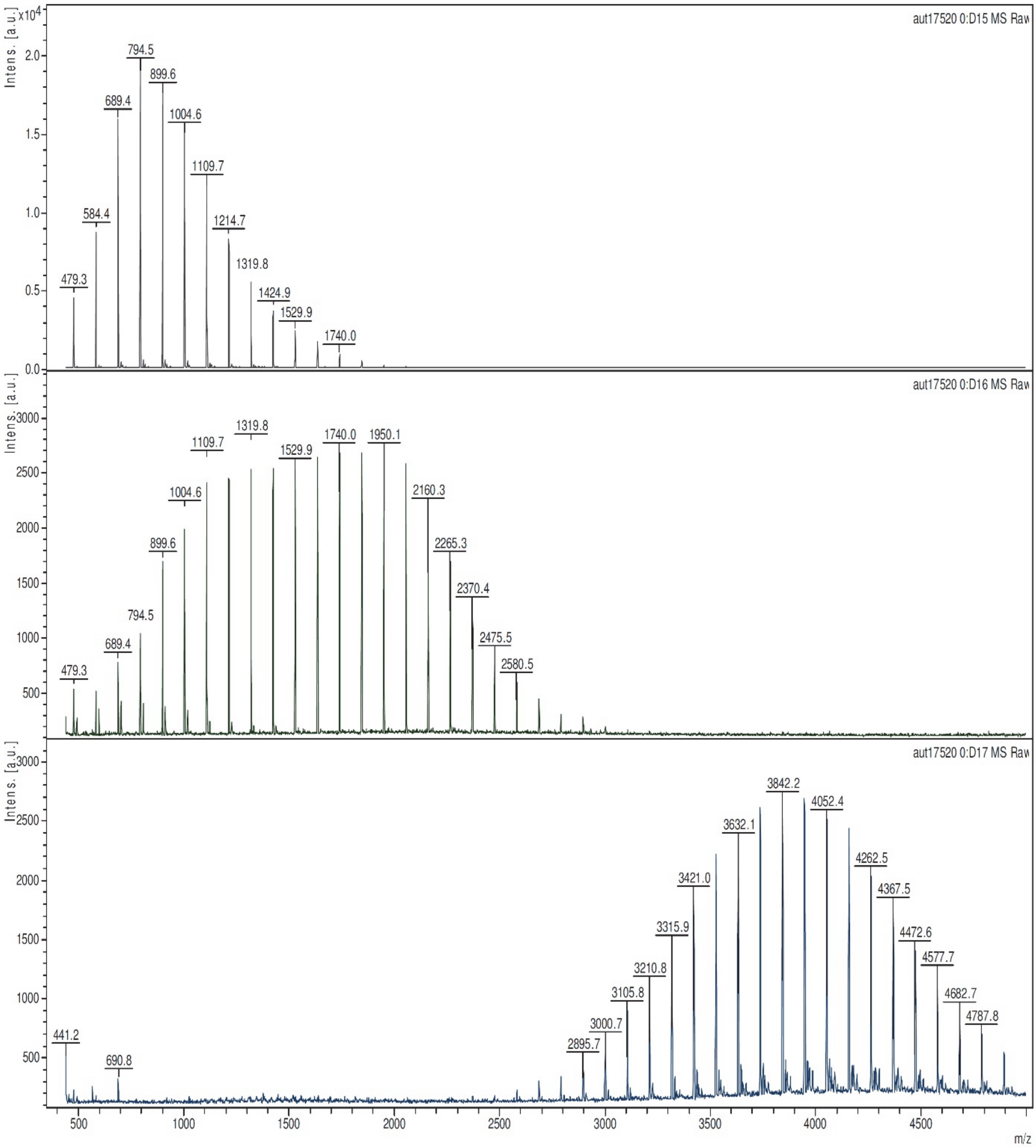

Fig. S3. Positive-ion linear mode MALDI-TOF spectra of PVP-067, PVP-1k, and PVP-2.1k (*top* to *bottom*) in dithranol matrix at laser power set to 35 %, 35%, and 40 %, respectively. All spectra are dominated by $[M+H]^+$ ions.

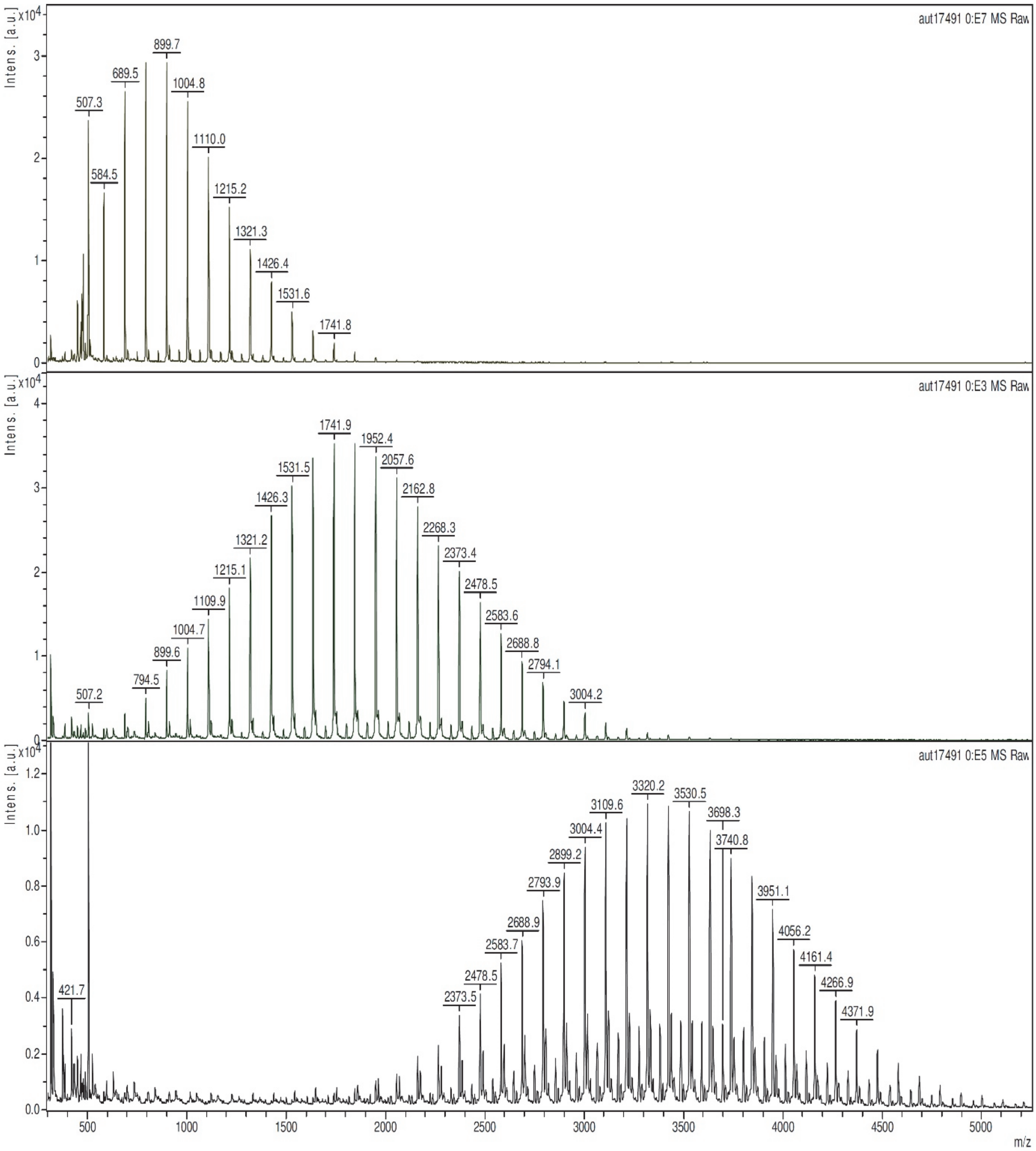

Fig. S4. Positive-ion reflector mode MALDI-TOF spectra of PVP-067, PVP-1k, and PVP-2.1k (*top* to *bottom*) in dithranol matrix. All spectra are dominated by $[M+H]^+$ ions.

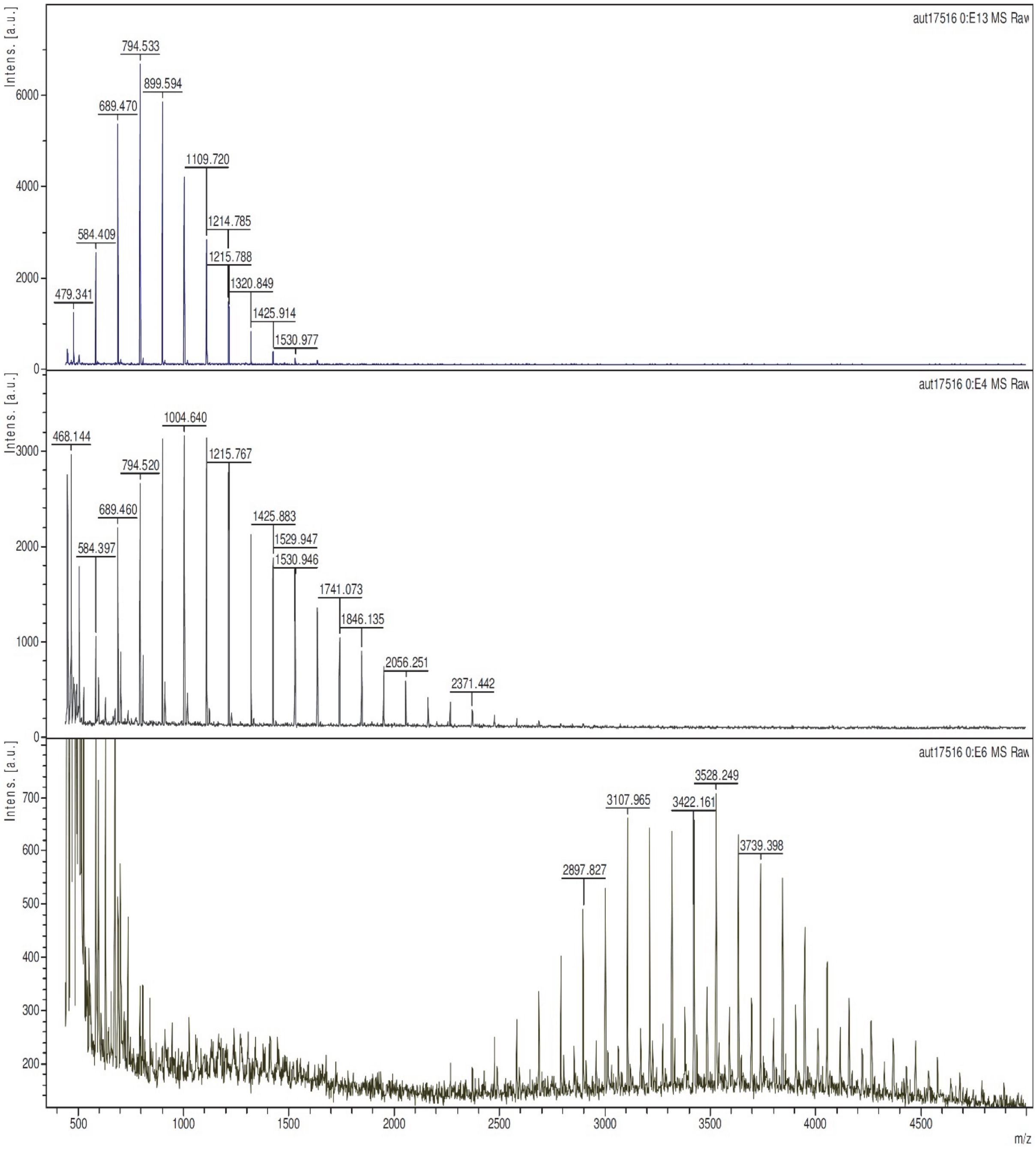

Fig. S5. Positive-ion linear mode MALDI-TOF spectra of PVP-067, PVP-1k, and PVP-2.1k (*top* to *bottom*) in 2,5-DHB matrix at laser power set to 35 %, 35%, and 40 %, respectively. All spectra are dominated by $[M+H]^+$ ions.

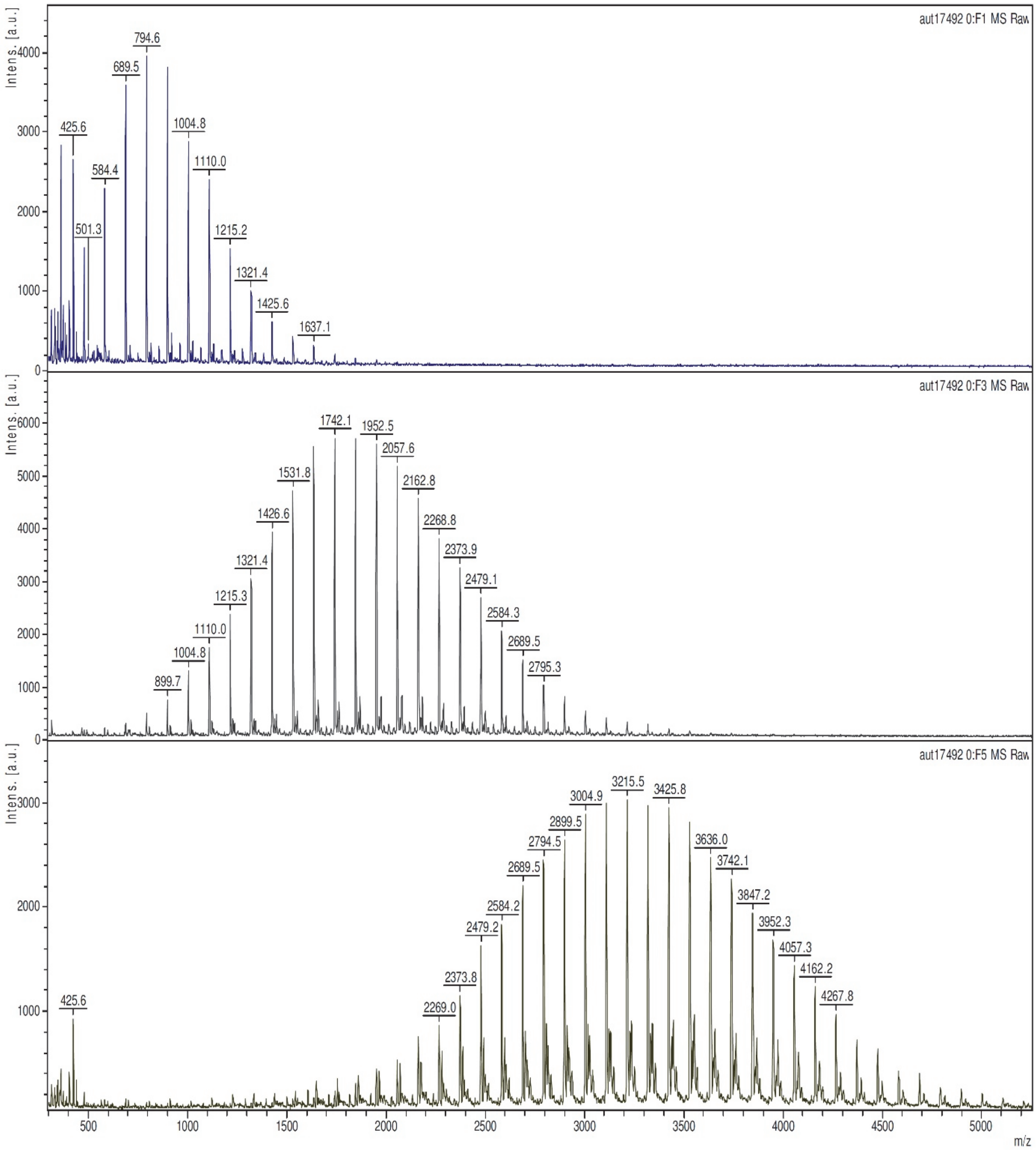

Fig. S6. Positive-ion reflector mode MALDI-TOF spectra of PVP-067, PVP-1k, and PVP-2.1k (*top* to *bottom*) in 2,5-DHB matrix. All spectra are dominated by $[M+H]^+$ ions.

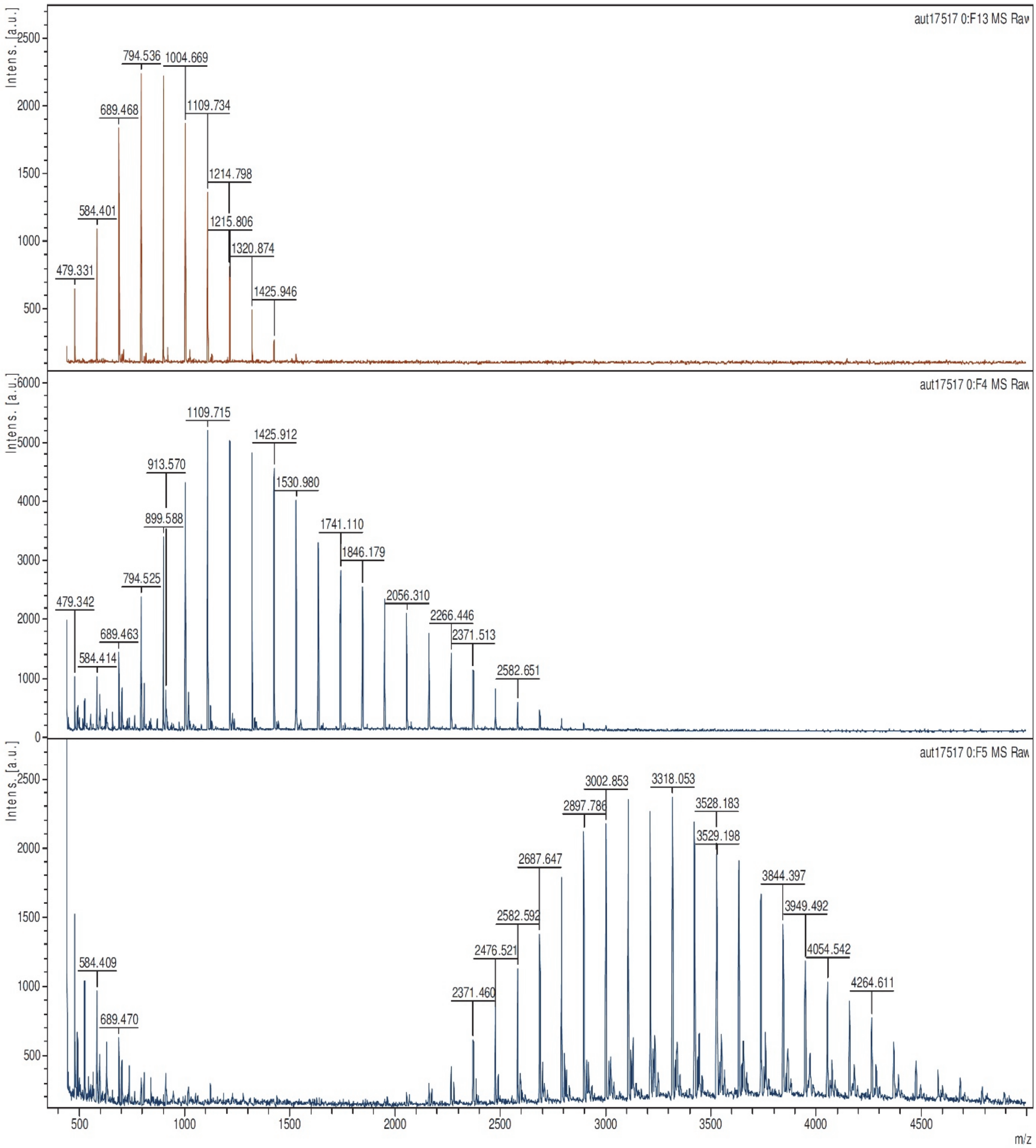

Fig. S7. Positive-ion linear mode MALDI-TOF spectra of PVP-067, PVP-1k, and PVP-2.1k (*top* to *bottom*) in DCTB matrix at laser power set to 15 %, 15%, and 20 %, respectively. All spectra are dominated by $[M+H]^+$ ions.

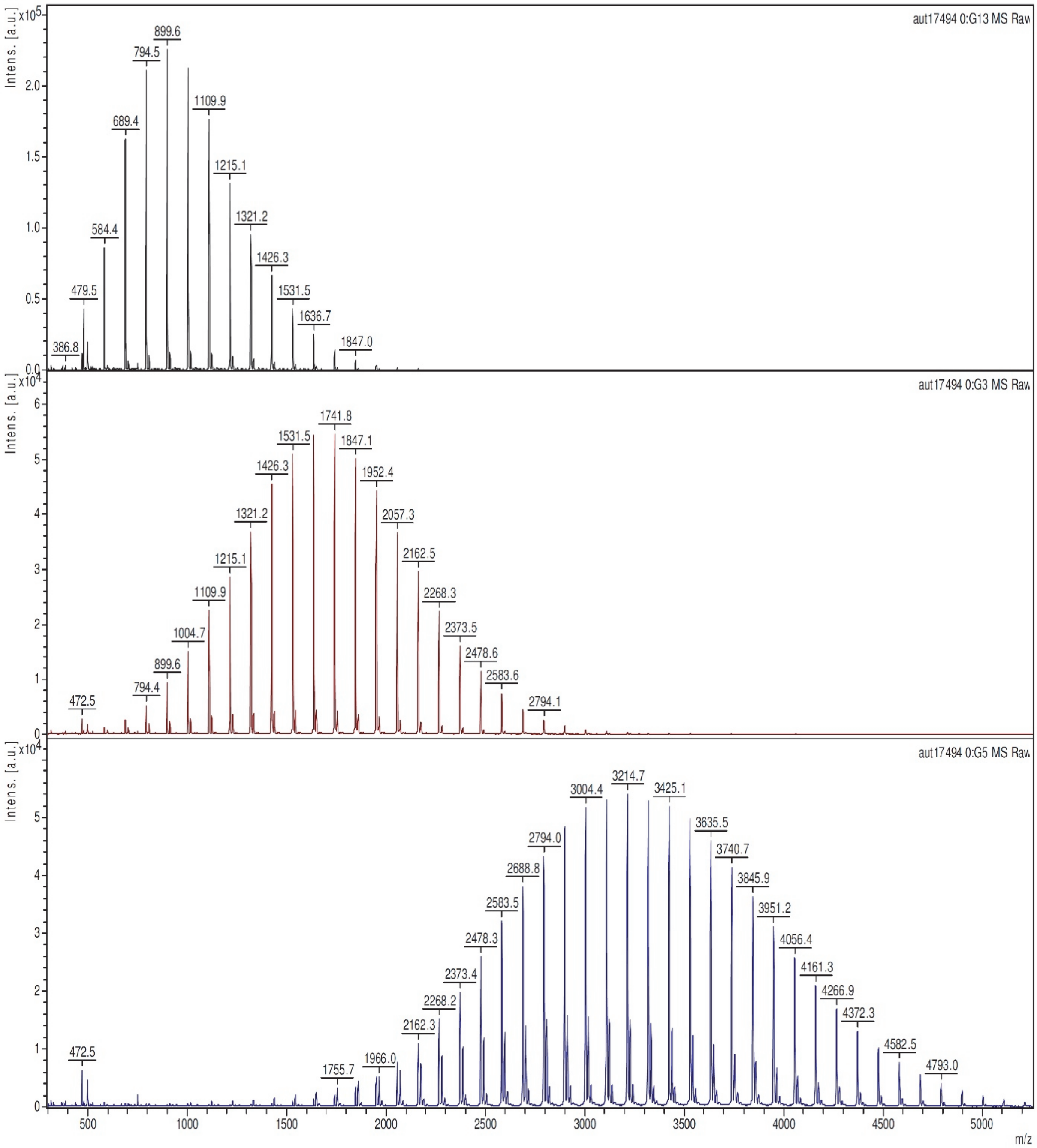

Fig. S8. Positive-ion reflector mode MALDI-TOF spectra of PVP-067, PVP-1k, and PVP-2.1k (*top* to *bottom*) in DCTB matrix. All spectra are dominated by $[M+H]^+$ ions.

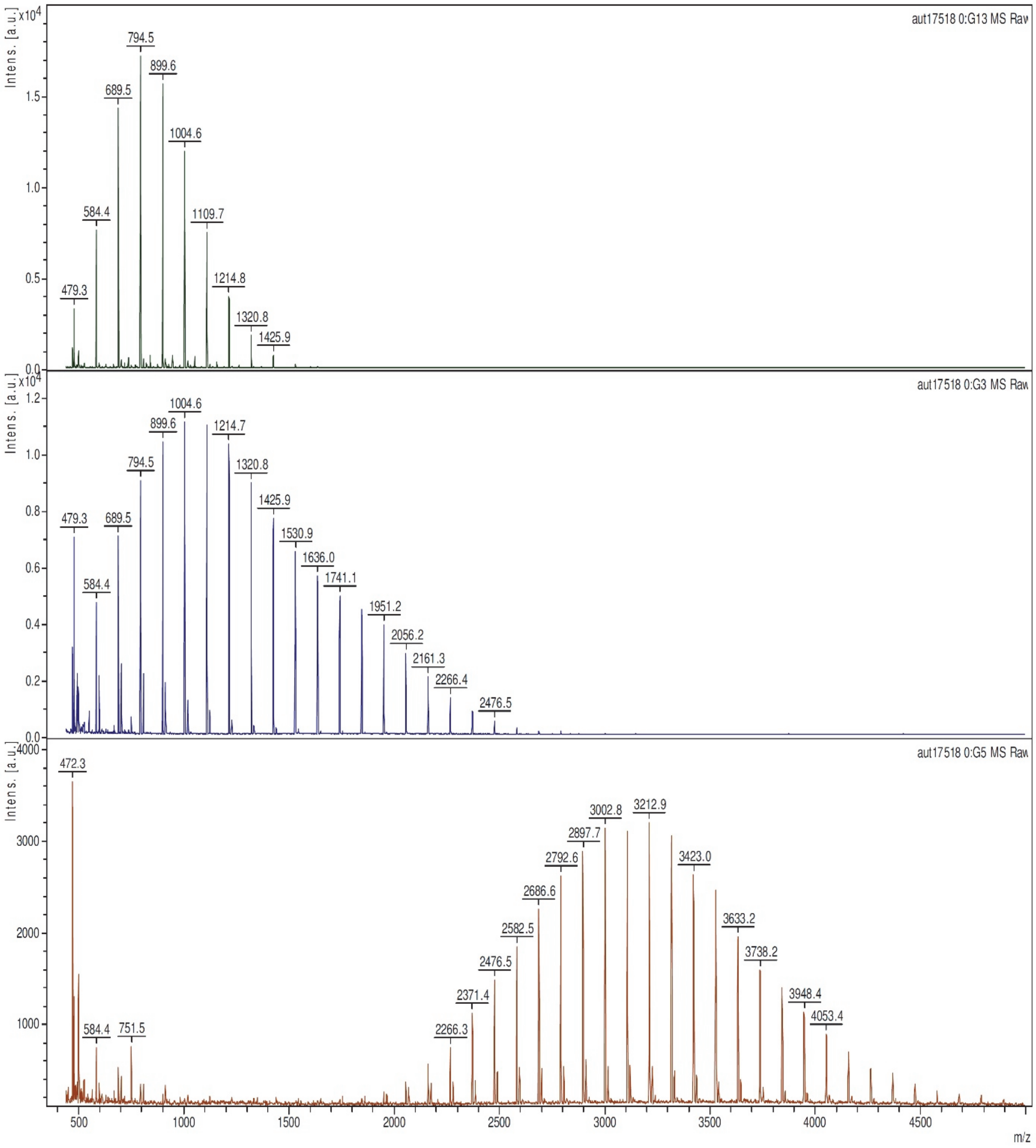

Fig. S9. Positive-ion linear mode MALDI spectra of PVP-1k in a) CHCA, b) dithranol), c) 2,5-DHB, and d) DCTB. For comparison of the relative responses all spectra were acquired with the laser power set to 25 %. The highest intensities in the center of the spectra approximate a) 2.5 x 10^5 a.u. (a.u.: arbitrary units of signal intensity), b) 5.0 x 10^3 a.u., c) 2.3 x 10^2 a.u., and d) 8.0 x 10^4 a.u., respectively. Any of the matrices yields mostly $[M+H]^+$ ions.

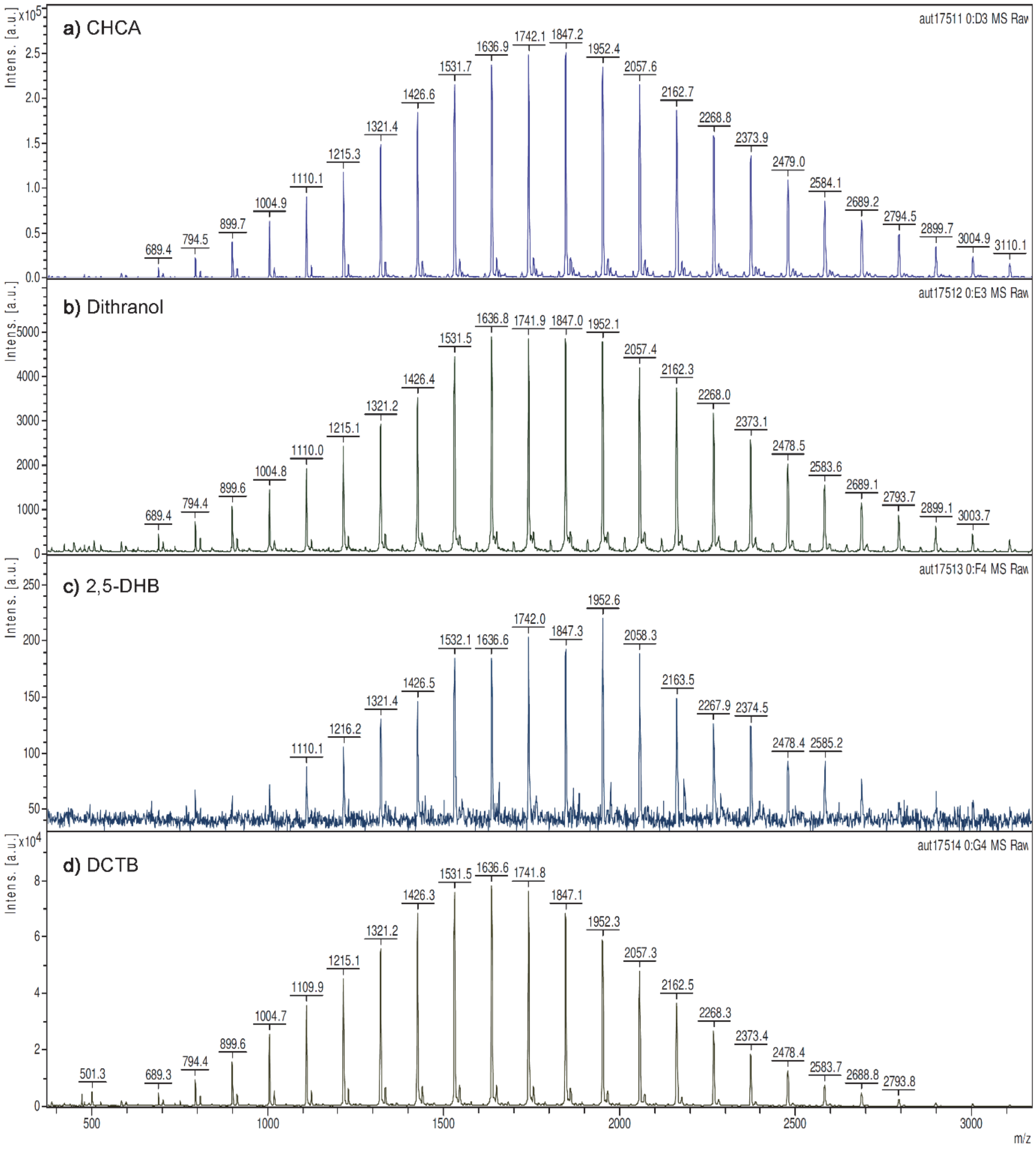

Fig. S10. Positive-ion reflector mode MALDI-TOF spectra of mixtures composed of PVP 067, PVP-1k, and PVP-2.1k as obtained in DCTB matrix. Examples show spectra of mixtures as required to cover different *m/z* ranges. In daily work, most used ranges were *m/z* 200–2000, *m/z* 340–3500, and *m/z* 440–4500(5000).

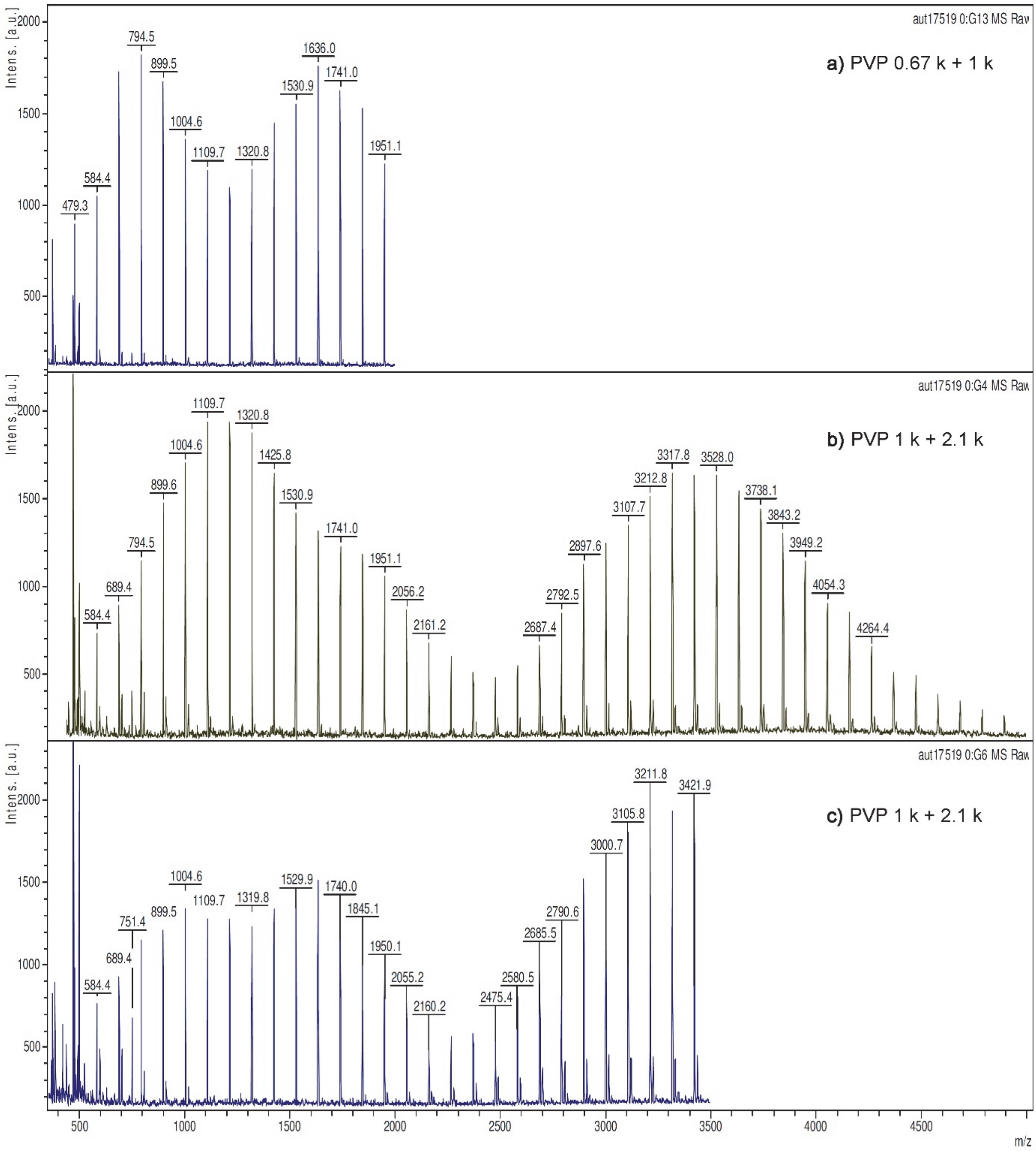

Fig. S11. Positive-ion DART-FT-ICR spectrum of PVP 067, 0.25 mg ml^{-1} in THF, 3 µl applied to OpenSpot card, DART gas helium at 450 °C, to confirm the formula assignment as $[M+H]^+$ ions. The accurate mass values from 4mer to 10mer along with proposed formulas are shown. The dots at the peak tips mark the monoisotopic ion signals used for formula assignment.

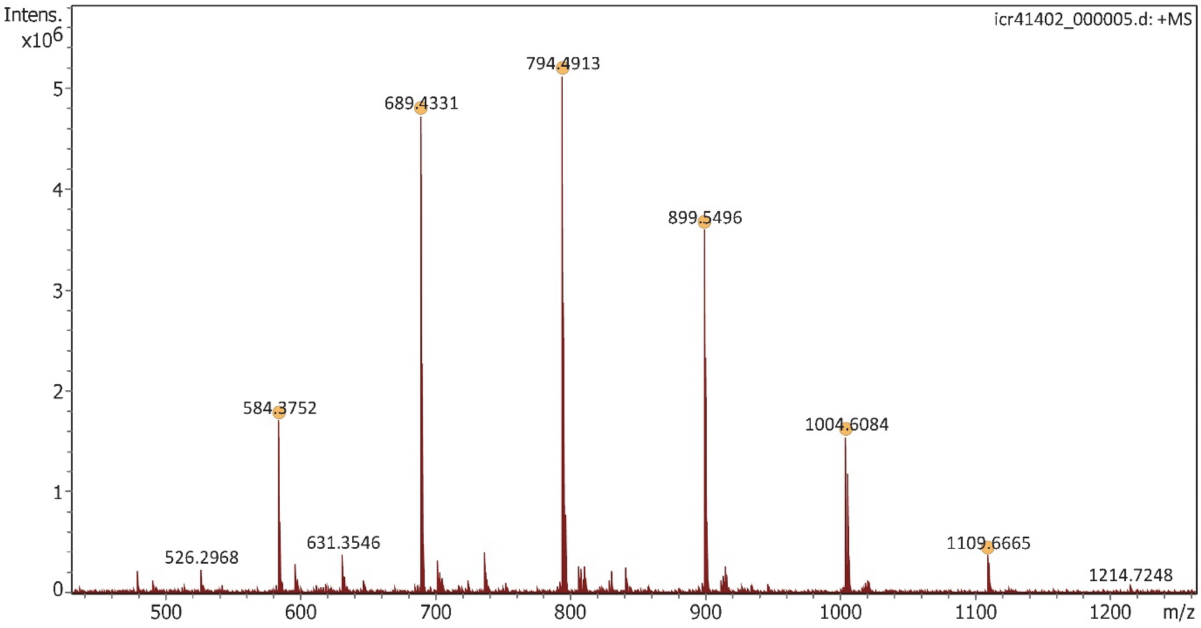

Meas. m/z	Ion Formula	m/z	err [ppm]	mSigma	rdb	e⁻ Conf	N-Rule
479.3175	C32H39N4	479.3169	-1.2	33.1	15.5	even	ok
584.3752	C39H46N5	584.3748	-0.6	25.6	19.5	even	ok
689.4331	C46H53N6	689.4326	-0.6	26.4	23.5	even	ok
794.4913	C53H60N7	794.4905	-1.1	27.1	27.5	even	ok
899.5496	C60H67N8	899.5483	-1.5	24.8	31.5	even	ok
1004.6084	C67H74N9	1004.6062	-2.2	23.4	35.5	even	ok
1109.6665	C74H81N10	1109.6640	-2.2	40.4	39.5	even	ok

Fig. S12. Positive-ion ESI-FT-ICR spectrum of PVP-1k in acetonitrile : water : THF = 4 : 2 : 1 with 0.1 % trifluoroacetic acid to confirm the formula assignment as $[M+H]^+$ ions. The accurate mass values along with proposed formulas are shown and correct PVP $[M+H]^+$ ionic compositions from 8mer to 18mer are marked in yellow. The dots at the peak tips mark the monoisotopic peaks used for formula assignment. The second series of ions on the left side with *m/z* 871.0109 and *m/z* 923.5392 among them was identified as doubly charged ions.

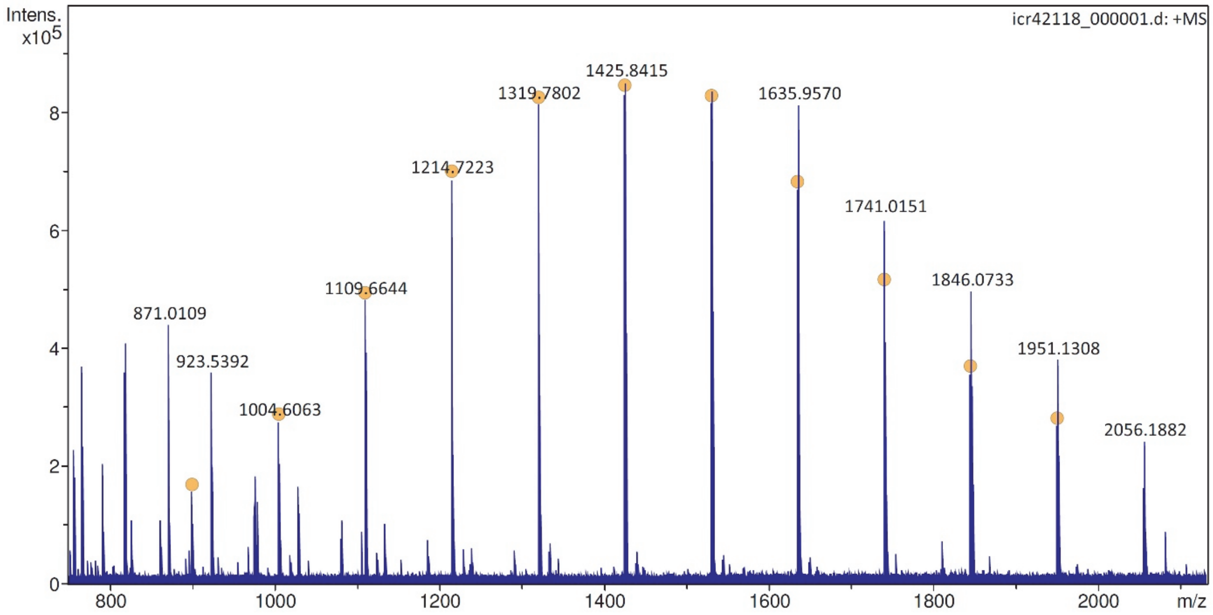

Meas. m/z	Ion Formula	m/z	err [ppm]	mSigma	rdb	e⁻ Conf	N-Rule
899.5475	C60H67N8	899.5483	0.9	26.4	31.5	even	ok
1004.6063	C67H74N9	1004.6062	-0.1	21.0	35.5	even	ok
1109.6644	C74H81N10	1109.6640	-0.4	19.6	39.5	even	ok
1214.7223	C81H88N11	1214.7219	-0.4	65.1	43.5	even	ok
1319.7802	C88H95N12	1319.7797	-0.4	56.3	47.5	even	ok
1424.8379	C95H102N13	1424.8376	-0.3	32.8	51.5	even	ok
	C99H235	1424.8383	0.3	38.3	-17.5	even	ok
1529.8955	C102H109N14	1529.8954	-0.0	54.3	55.5	even	ok
	C106H242N	1529.8962	0.5	59.1	-13.5	even	ok
1634.9536	C109H116N15	1634.9533	-0.2	34.9	59.5	even	ok
	C113H249N2	1634.9540	0.3	38.4	-9.5	even	ok
1740.0116	C116H123N16	1740.0111	-0.3	31.2	63.5	even	ok
	C120H256N3	1740.0119	0.2	34.3	-5.5	even	ok
1845.0698	C123H130N17	1845.0690	-0.5	18.7	67.5	even	ok
	C127H263N4	1845.0697	-0.1	20.3	-1.5	even	ok
	C153H9	1845.0699	0.0	90.2	149.5	even	ok
1950.1269	C130H137N18	1950.1268	-0.0	70.9	71.5	even	ok
	C134H270N5	1950.1276	0.4	73.8	2.5	even	ok
	C160H16N	1950.1277	0.4	138.7	153.5	even	ok

Fig. S13. Positive-ion MALDI-Q-TOF spectra of **a)** a mixture composed of PVP 067 and PVP-2.1k 1 : 3, **b)** PVP670, **c)** PVP-1k, and **d)** PVP2.1k as obtained in DCTB matrix using the Bruker timsTOF flex instrument. The appearance of the PVP spectra is almost identical to those obtained using the axial TOF in reflector mode. Spectrum **a)** is used for mass calibration (cf. calibration report in Fig. S14 for *m/z* values assigned to the peaks).

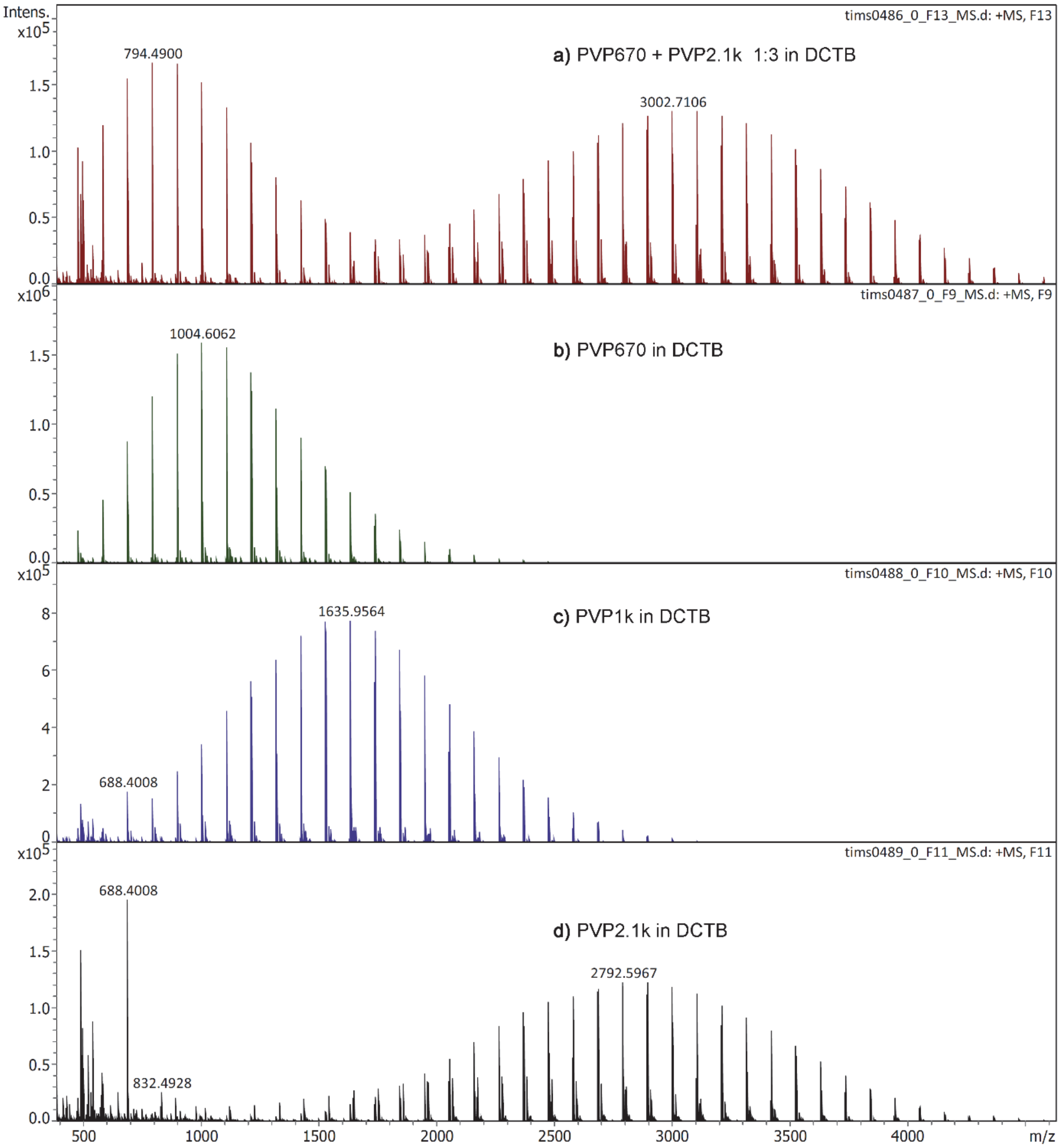

Fig. S14. Mass calibration report after calibration of the positive-ion MALDI-Q-TOF spectrum (Bruker timsTOF flex instrument) of a mixture composed of PVP 067 and PVP-2.1k 1 : 3 (cf. a) in Fig. S13) in DCTB matrix. The average error of calibrant ion m/z values is 0.277 ppm.

Calibration Report

Analysis Info

Analysis	D:\Data\Gross\tims0539_0_F21_MS.d	Acquisition Date	8/27/2021 8:02:46 AM
Method	MALDIpos_250-5000.m	Operator	TOF-User
Sample	tims0539	Instrument	timsTOF fleX 1859745.20462
Comment	PVP 067 + 2.1k 1:3 in DCTB, 1-3 13C reflist		

Acquisition Parameter

Source Type	ESI	Ion Polarity	Positive	Laser Power	20 %
Scan Begin	250 m/z	Set Capillary	0 V	Laser Shots	8000
Scan End	5000 m/z	Set Multipole RF	480.0 Vpp	Laser Application	MS Dried Droplet
		Set Collision Cell RF	1800.0 Vpp	Beam Scan	On
		IMS Active	Off	Shots/Position	200
		IMS Collision Cell In	300.0 V		

Calibration Status

Initial calibration from acquisition

Date:	8/27/2021 8:01:34 AM
Polarity:	Positive
Calibration spectrum:	<unknown>
Reference mass list:	PVP-polyvinylpyridin-pos13C_1-3
Calibration mode:	Enhanced Quadratic
Standard deviation:	0.277 ppm

Reference m/z	Resulting m/z	Intensity	Error [ppm]
269.2012	269.2014	26664	0.647
479.3169	479.3167	247873	-0.482
584.3748	584.3746	309415	-0.366
689.4326	689.4324	425474	-0.252
794.4905	794.4903	464194	-0.152
899.5483	899.5482	477561	-0.122
1004.6062	1004.6061	428193	-0.093
1109.6640	1109.6640	368900	0.017
1214.7219	1214.7221	288743	0.177
1320.7831	1320.7831	213214	0.049
1425.8409	1425.8409	165395	0.004
1530.8988	1530.8987	120107	-0.036
1635.9566	1635.9568	87797	0.100
1741.0145	1741.0145	62498	0.018
1846.0723	1846.0731	48858	0.401
1951.1302	1951.1306	41650	0.237
2056.1880	2056.1888	39389	0.365
2161.2459	2161.2468	40375	0.424
2266.3037	2266.3039	44728	0.091
2371.3616	2371.3619	46907	0.158
2476.4194	2476.4198	53971	0.158
2581.4773	2581.4774	57364	0.060
2687.5385	2687.5384	58940	-0.036
2792.5963	2792.5960	61511	-0.108
2897.6542	2897.6536	62411	-0.205
3002.7120	3002.7108	61987	-0.392
3107.7699	3107.7695	59653	-0.125
3212.8277	3212.8268	54540	-0.283
3317.8856	3317.8849	49945	-0.208
3422.9434	3422.9418	42304	-0.467
3528.0013	3528.0010	35145	-0.071
3633.0591	3633.0584	28759	-0.202
3738.1170	3738.1166	21754	-0.094
3843.1748	3843.1755	15656	0.169
3948.2327	3948.2320	11699	-0.160
4053.2905	4053.2904	7023	-0.039
4159.3517	4159.3521	5087	0.088
4264.4096	4264.4099	3131	0.078
4369.4674	4369.4681	1737	0.155
4474.5253	4474.5290	875	0.841
4579.5831	4579.5815	586	-0.347

Initial mobility calibration from acquisition

Bruker Compass DataAnalysis 5.3 printed: 8/27/2021 8:03:46 AM by: demo Page 1

Table S3. Mass accuracy by MALDI-FT-ICR-MS based on external calibration. Data of PEG 1000 $[M+Na]^+$ ions, five repetitions, standard deviation of *m/z* in ppm, average error $\Delta(m/z)$ in mu. The accuracy level is around 2 ppm.

Formula	Calc. *m/z*	Exp. *m/z*	Exp. *m/z*	Exp. *m/z*	Exp. *m/z*	Exp. *m/z*	Avg. Exp. *m/z*	Std. Dev.
$[C36H74O19Na]^+$	833.4717	833.4699	833.4699	833.4693	833.4699	833.4699	833.4698	0.3
$\Delta(m/z)$		0.0018	0.0018	0.0024	0.0018	0.0018	0.0019	2.2
$[C38H78O20Na]^+$	877.4979	877.4955	877.4964	877.4962	877.4954	877.4968	877.4961	0.7
$\Delta(m/z)$		0.0024	0.0015	0.0017	0.0025	0.0011	0.0018	2.1
$[C40H82O21Na]^+$	921.5241	921.5212	921.5223	921.5216	921.5221	921.5225	921.5219	0.6
$\Delta(m/z)$		0.0029	0.0018	0.0025	0.0020	0.0016	0.0021	2.3
$[C42H86O22Na]^+$	965.5503	965.5476	965.5484	965.5483	965.5473	965.5491	965.5481	0.7
$\Delta(m/z)$		0.0027	0.0019	0.0020	0.0030	0.0012	0.0022	2.2
$[C44H90O23Na]^+$	1009.5765	1009.5735	1009.5743	1009.5736	1009.5742	1009.5745	1009.5740	0.4
$\Delta(m/z)$		0.0030	0.0022	0.0029	0.0023	0.0020	0.0025	2.5
$[C46H94O24Na]^+$	1053.6027	1053.5994	1053.6004	1053.6005	1053.5995	1053.6013	1053.6002	0.7
$\Delta(m/z)$		0.0033	0.0023	0.0022	0.0032	0.0014	0.0025	2.4
$[C48H98O25Na]^+$	1097.6289	1097.6256	1097.6266	1097.6264	1097.6259	1097.6273	1097.6264	0.6
$\Delta(m/z)$		0.0033	0.0023	0.0025	0.0030	0.0016	0.0026	2.4
$[C50H102O26Na]^+$	1141.6552	1141.6514	1141.6523	1141.6518	1141.6520	1141.6527	1141.6520	0.4
Delta		0.0038	0.0029	0.0034	0.0032	0.0025	0.0031	2.7
$[C52H106O27Na]^+$	1185.6814	1185.6776	1185.6787	1185.6782	1185.6782	1185.6795	1185.6784	0.6
$\Delta(m/z)$		0.0038	0.0027	0.0032	0.0032	0.0019	0.0029	2.5
$[C54H110O28Na]^+$	1229.7076	1229.7036	1229.7048	1229.7044	1229.7039	1229.7058	1229.7045	0.7
$\Delta(m/z)$		0.0040	0.0028	0.0032	0.0037	0.0018	0.0031	2.5
$[C56H114O29Na]^+$	1273.7338	1273.7294	1273.7308	1273.7302	1273.7303	1273.7317	1273.7305	0.7
$\Delta(m/z)$		0.0044	0.0030	0.0036	0.0035	0.0021	0.0033	2.6
$[C58H118O30Na]^+$	1317.7600	1317.7549	1317.7561	1317.7549	1317.7561	1317.7563	1317.7557	0.5
$\Delta(m/z)$		0.0051	0.0039	0.0051	0.0039	0.0037	0.0044	3.3
$[C60H122O31Na]^+$	1361.7862	1361.7811	1361.7828	1361.7814	1361.7826	1361.7838	1361.7823	0.8
$\Delta(m/z)$		0.0051	0.0034	0.0048	0.0036	0.0024	0.0039	2.9
$[C62H126O32Na]^+$	1405.8124	1405.8069	1405.8088	1405.8087	1405.8075	1405.8106	1405.8085	1.0
$\Delta(m/z)$		0.0055	0.0036	0.0037	0.0049	0.0018	0.0039	2.8
$[C64H130O33Na]^+$	1449.8387	1449.8334	1449.8352	1449.8338	1449.8342	1449.8355	1449.8344	0.6
$\Delta(m/z)$		0.0053	0.0035	0.0049	0.0045	0.0032	0.0042	2.9
$[C66H134O34Na]^+$	1493.8649	1493.858	1493.8599	1493.8607	1493.8585	1493.8623	1493.8599	1.2
$\Delta(m/z)$		0.0069	0.0050	0.0042	0.0064	0.0026	0.0050	3.3

Fig. S15. Positive-ion MALDI-FT-ICR spectrum of PEG 1000 in DCTB matrix. This is one of the spectra used in Table S3.

**Analysis Info**		Acquisition Date	23.02.2021 11:00:27
Analysis Name	Z:\Gross\icr41684_0_F19_000002.d		
Comment	PEG1000 extern mit PVP 0.67 + 2.1, 1:2, 5 mg/ml 1:50 in DCTB		

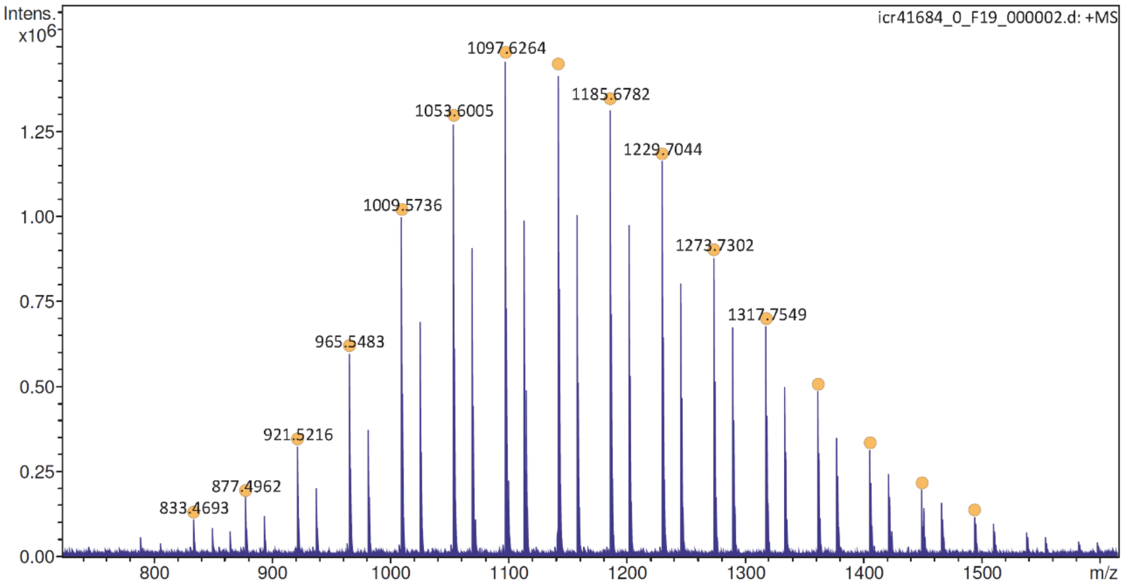

Meas. m/z	Ion Formula	m/z	err [ppm]	mSigma	rdb	e⁻ Conf	N-Rule
833.4693	C36H74NaO19	833.4717	2.9	17.9	-0.5	even	ok
877.4962	C38H78NaO20	877.4979	1.9	29.1	-0.5	even	ok
921.5216	C40H82NaO21	921.5241	2.7	57.9	-0.5	even	ok
965.5483	C42H86NaO22	965.5503	2.1	32.0	-0.5	even	ok
1009.5736	C44H90NaO23	1009.5765	2.9	23.5	-0.5	even	ok
	C51H86NaO18	1009.5706	-2.9	52.7	8.5	even	ok
1053.6005	C46H94NaO24	1053.6027	2.2	29.6	-0.5	even	ok
1097.6264	C48H98NaO25	1097.6289	2.3	26.2	-0.5	even	ok
	C55H94NaO20	1097.6231	-3.1	61.0	8.5	even	ok
1141.6518	C50H102NaO26	1141.6552	2.9	28.1	-0.5	even	ok
	C57H98NaO21	1141.6493	-2.2	57.0	8.5	even	ok
1185.6782	C52H106NaO27	1185.6814	2.7	29.9	-0.5	even	ok
	C59H102NaO22	1185.6755	-2.2	65.4	8.5	even	ok
1229.7044	C54H110NaO28	1229.7076	2.6	32.0	-0.5	even	ok
	C61H106NaO23	1229.7017	-2.2	68.4	8.5	even	ok
1273.7302	C56H114NaO29	1273.7338	2.8	32.4	-0.5	even	ok
	C63H110NaO24	1273.7279	-1.8	68.0	8.5	even	ok
1317.7549	C65H114NaO25	1317.7541	-0.6	63.5	8.5	even	ok
1361.7814	C67H118NaO26	1361.7804	-0.8	70.5	8.5	even	ok
1405.8087	C62H126NaO32	1405.8124	2.7	14.0	-0.5	even	ok
	C69H122NaO27	1405.8066	-1.5	43.7	8.5	even	ok
1449.8338	C71H126NaO28	1449.8328	-0.7	44.5	8.5	even	ok
	C89H118NaO15	1449.8363	1.7	138.2	30.5	even	ok
1493.8607	C73H130NaO29	1493.8590	-1.1	68.0	8.5	even	ok
	C91H122NaO16	1493.8625	1.2	138.1	30.5	even	ok
